# Ecological Momentary Assessment to Measure Social Connectedness in Older Adults: Integrative Review

**DOI:** 10.2196/66324

**Published:** 2025-06-17

**Authors:** Seongmi Choi, Hun Kang, Jiyoung Shin, Sang Hui Chu, JiYeon Choi

**Affiliations:** 1 Research Institute of Nursing Science, Daegu Catholic University College of Nursing Daegu Republic of Korea; 2 Mo-Im Kim Nursing Research Institute, Yonsei University College of Nursing Seoul Republic of Korea; 3 Yonsei University College of Nursing Seoul Republic of Korea; 4 Health Insurance Research Institute, National Health Insurance Service Gangwon-Do Republic of Korea; 5 Institute for Innovation in Digital Healthcare, Yonsei University Seoul Republic of Korea

**Keywords:** aged, social connectedness, ecological momentary assessment, review, mobile phone

## Abstract

**Background:**

The importance of social connectedness as a determinant of health and well-being in older adults is well-established. Ecological momentary assessment (EMA) shows promise for real-time measurement of social interactions, making it worthwhile to investigate its feasibility and the challenges of applying it to older adults.

**Objective:**

This integrative review aimed to (1) summarize and integrate the implementation of EMA in assessing older adults’ social connectedness, and (2) discuss the EMA method and its use to assess the concept of social connectedness in order to guide future research.

**Methods:**

A total of 5 databases—PubMed, CINAHL, Embase, Web of Science, and PsycINFO—were searched for studies published up to March 2025. We included studies that (1) targeted adults aged 60 years or older, (2) used EMA to assess social connectedness, and (3) were published in a peer-reviewed journal. Studies using third-party reports to obtain EMA data and studies focusing on marital dyads were excluded. The analysis identified multifactorial constructs of social connectedness (structural, functional, and quality) and assessed EMA protocols and compliance or adherence to EMA.

**Results:**

Of the 18,886 studies identified, 43 were selected for final analysis. Social connectedness assessed via EMA mostly focused on the structural dimension, capturing whether an individual had social contact at a given moment (38/43, 88%). Among functional dimension (17/43, 40%), loneliness was the most measured construct, and the quality dimension (16/43, 37%) included quality of social interaction, pleasantness of encounters, and interpersonal tensions. In total, 2 studies addressed all 3 dimensions of social connectedness. In addition, to provide context for understanding social connectedness, assessments considered location at the time of assessment, type of activity, and physical (eg, pain and fatigue) and psychological states (eg, positive or negative mood). Data were mostly collected using an app on digital devices (eg, smartphone), and assessments were conducted 1-7 times per day for 5 to 25 days, achieving a compliance rate of over 70%.

**Conclusions:**

The findings of this study highlight the current state of science in measuring social connectedness in older adults through EMA and demonstrate its feasibility in real-world settings. Further research is suggested to address the conceptual and methodological challenges of EMA, as measurement of multifactorial constructs of social connectedness and standardization of EMA protocols may increase the likelihood of capturing useful information about older adults’ real-time social connectedness.

**Trial Registration:**

PROSPERO CRD42024499050; https://www.crd.york.ac.uk/PROSPERO/view/CRD42024499050

## Introduction

Social connectedness is a complex phenomenon encompassing numerous emotional, physical, and behavioral aspects of human interaction, and it is recognized as an important contributor to individual and population health and well-being [1]. In older adults, a lack of social connectedness has been linked to adverse effects on a broad spectrum of health outcomes, such as depression, cardiovascular disease, quality of life, overall health, cognitive function, and mortality [2]. Although aging itself does not directly cause a reduction in social connectedness, older adults often face an increased prevalence of loss, changes in functional independence, frailty, declining health status, deteriorating relationship quality, shifts in care needs and living arrangements, changes in employment status, and financial instability. These factors can make maintaining social connections more challenging [3-6]. As the world’s population is expected to age [7], the importance of social connectedness is highlighted regarding its role in optimizing the physical health, psychosocial health, and well-being of older adults.

Despite the well-acknowledged importance of social connectedness, terms such as social contact, integration, perceived support, and loneliness are often used interchangeably [8]. However, these terms do not fully capture the breadth of the concept [9]. To address this issue, social connectedness has been conceptualized as an umbrella term that encompasses various dimensions, categorized into structural, functional, and quality dimensions [8]. Given that each dimension independently influences health outcomes and that the correlations between them are weak, it is crucial to analyze them separately to understand their distinct pathways to health [10].

Ecological momentary assessment (EMA), or experience sampling method, measures individuals’ daily experiences in real time, minimizing researcher control and collecting data in natural settings. This approach helps capture participants’ momentary, specific daily experiences. EMA typically uses mobile devices (eg, smartphones and digital wristwatches) to prompt participants to answer short, targeted questions multiple times a day over several days or weeks [11,12]. While retrospective recall has been the primary method for assessing social connectedness, it is prone to bias and errors, particularly for minor events like everyday social interactions [11,13]. In contrast, EMA minimizes recall bias and provides detailed, real-time insights into social dynamics [14,15].

However, the feasibility and usability of assessing social connectedness in older adults using EMA remain underexplored. Previous studies have primarily focused on younger populations, often overlooking the unique challenges that older adults may encounter, particularly in relation to technology use [16-18]. There are several reviews that highlight the feasibility and application of employing EMA in an aging population [13,19,20]. However, no known study has focused on evaluating EMA as a tool to assess social connectedness. Identifying practical challenges and opportunities associated with EMA in studying social connectedness among older adults will provide valuable methodological insights that can support future research. To address this gap, we conducted an integrative review, a research approach that synthesizes findings from diverse methodologies to provide a comprehensive understanding of the phenomenon of interest [21]. Accordingly, this study aimed to (1) summarize and integrate the use of EMA in evaluating social connectedness among older adults, and (2) discuss the concept of social connectedness as assessed by EMA, as well as the EMA methodology, to guide future research.

## Methods

### Search Design

This integrative review was based on the methodology proposed by Whittemore and Knafl [21]. The protocol for this review was registered with PROSPERO (CRD42024499050). Two deviations from the registered protocol were made. First, we collected more comprehensively relevant studies by including additional search terms related to EMA. Second, we extended the literature search period beyond the planned period to include up-to-date studies. This review was guided by the PRISMA (Preferred Reporting Items for Systematic Reviews and Meta-Analyses) [22]. The PRISMA checklist was provided as a Multimedia Appendix 1.

This review focused on the following variables of interest: target population (older adults aged 60 years or older), concept (EMA), and context (social connectedness). In this study, EMA was defined as a method of repeatedly measuring experiences, behaviors, and emotions in real time or in close proximity to an individual’s daily environment. EMA has properties of repeated measurements such as prompt type (eg, time-based and event-based), collection period (within or over several days), collection frequency within a day, and data collection tools were set as a comprehensive concept that includes paper-based questionnaires and methods using digital technologies such as smartphones, computers, and wearable devices. Social connectedness is defined as a concept encompassing the structural (eg, social contact), functional (eg, loneliness), and quality dimensions (eg, relationship strain) of an individual’s experience through social relationships [8].

### Search Strategies

We conducted a systematic search to identify relevant studies published from the inception of the databases up to March 2025. This study included 5 electronic databases—PubMed, CINAHL, Embase, Web of Science, and PsycINFO. The search terms were developed using a combination of MeSH (Medical Subject Headings) terms and keywords, with the assistance of a professional medical librarian. We have summarized the combination of search terms used for each database in Multimedia Appendix 2.

### Eligibility Criteria

The inclusion criteria for the studies were (1) the participants were adults aged 60 years or older, (2) the studies used EMA to evaluate social connectedness, and (3) the studies were published in English. The exclusion criteria included (1) case reports, reviews, editorials, descriptive commentary, conference abstracts, unpublished master’s theses, and doctoral dissertations; (2) studies not specifically targeting older adults; (3) studies that relied on third-party reports to collect EMA data; and (4) studies focusing exclusively on marital dyads. Studies that rely on third-party reports to collect real-time data in clinical settings (eg, patients with dementia residing in long-term care facilities) introduce subjectivity and are limited in their ability to reflect real-world scenarios, as not all situations can be directly observed. Furthermore, although marital dyads can significantly influence the social connectedness of older adults [23,24], they were excluded from this review due to their focus on a specific type of social relationship.

### Study Screening and Selection

For screening and selection, all studies were imported into Microsoft Excel. Duplicate records were identified and manually removed within Excel based on matching titles, authors, and publication years. Two authors (SC and HK) independently screened the titles and abstracts of all studies using predefined eligibility criteria. After excluding irrelevant studies, each author independently reviewed the full texts of the remaining studies. At each stage, following the independent screening process, the authors discussed any discrepancies and reached a consensus on the eligibility of each study. These authors had an agreement on the final selection of the studies.

### Quality Evaluation of the Selected Studies

Two authors, SC and HK, independently assessed the quality of the selected studies using the 2018 version of the Mixed-Methods Appraisal Tool (MMAT) [25]. The MMAT is designed to evaluate various study designs, including quantitative, qualitative, and mixed methods, using distinct quality criteria for each type. The tool is divided into 2 parts; part 1, which applies uniform screening criteria across all study designs, and part 2, which uses criteria specific to each design. The items used in part 2 include the following: (1) Are the participants representative of the target population? (2) Are measurements appropriate regarding both the outcome and intervention (or exposure)? (3) Are there complete outcome data? (4) Are the confounders accounted for in the design and analysis? (5) During the study period, is the intervention administered (or exposure occurred) as intended? Responses in part 2 are categorized as “yes,” “no,” or “cannot tell.” We reported the overall quality scores with asterisks, ranging from “none” (none of the quality criteria were met) to “*****” (all 5 criteria were met) [25].

### Data Extraction and Synthesis

Data were extracted using a standardized Microsoft Excel form. Two authors (SC and HK) extracted the selected data into an analysis table. These authors validated and confirmed the analyzed data between articles and table entries for accuracy. Data extracted from studies selected for final review included study characteristics such as author, year of publication, region where the study was conducted, study purpose, sample (size and age), data source, main findings, and factors associated with social connectedness. In addition, EMA protocol details and adherence or compliance rates were reviewed and extracted for each study. Methodological elements—such as prompt design, definition of moment, sampling frequency, device type, training, response strategies, and criteria for valid responses—were extracted. Each study was categorized by data source and compared by identifying the EMA protocol and adherence or compliance rate. To avoid overinterpretation, the findings were synthesized at the data source level.

To determine the comprehensiveness of social connectedness assessed using EMA in the selected studies, we used the framework by Holt-Lunstad et al [8] that depicted indicators of social connectedness with 3 dimensions, that is, structure, function, and quality. The structural dimension is generally quantitative, evaluating the number or diversity of social relationships, or the frequency of social contact (eg, social network and social contact). The functional dimension assesses the actual or perceived availability of support and resources that relationships can provide (eg, perceived social support and perceived loneliness). The quality dimension reflects perceptions of the positive and negative aspects of social relationships (eg, relationship strain and marital quality) [8]. Each dimension was divided into trait-level assessment and EMA, with the EMA further categorized according to the definition of moment.

## Results

### Characteristics of the Included Studies

**Figure 1:**
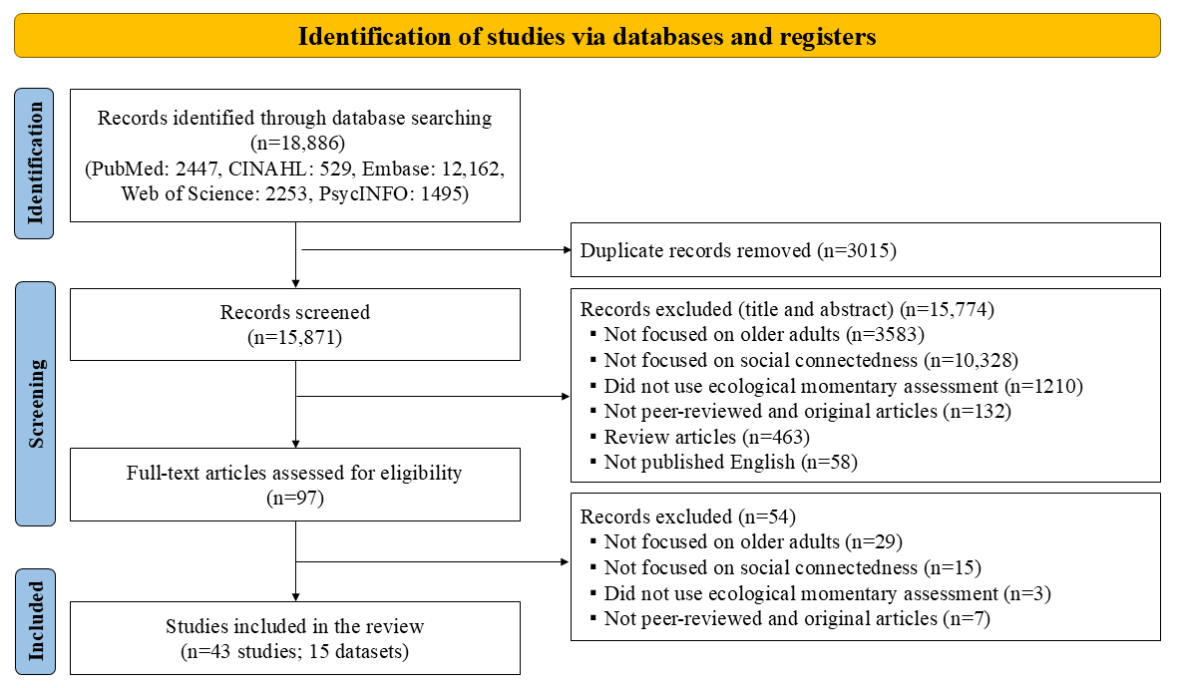
PRISMA (Preferred Reporting Items for Systematic Reviews and Meta-Analyses) flow diagram outlining the search and review process.

Figure 1 presents a summary of the literature search and selection process using the PRISMA flow diagram. Initially, 18,886 studies were identified across 5 databases; 2447 from PubMed, 529 from CINAHL, 12,162 from Embase, 2253 from Web of Science, and 1495 from PsycINFO. After eliminating 3015 duplicates, we screened the titles and abstracts of 15,871 studies. Subsequently, 97 studies underwent a full-text review. The final sample included 43 studies based on 15 distinct datasets.

Multimedia Appendix 3 provides a summary of the characteristics of the included studies, which were published between 2001 and 2024. Most studies were conducted in the United States (34/43, 79%), with 7 studies from Switzerland (7/43, 16%), 1 each from Australia (1/43, 2%), Canada, and Hong Kong (1/43, 2%). All studies were quantitative in nature and designed with a prospective approach. Most studies used data from large-scale projects such as the Daily Experiences and Well-being Study [26-40], the Einstein Aging Study [15,41-47], the Chicago Health and Activity Space in Real-Time (CHART) study [48-51], and the study on digitalization and social lives of older adults [52-56]. These studies primarily assessed the daily experiences, health, and well-being of older adults. All but 2 studies [57,58] that targeted the general adult population included community-dwelling older adults aged 60 years or older, with sample sizes ranging from 173 to 477. The 2 studies that included the entire adult population showed age-specific characteristics by categorizing them into young, middle, and older adults, with the cutoff for older adults being 60 years or older [58] and 65 years or older [57].

Among the variables mainly used as outcomes of social connectedness, loneliness accounted for the largest proportion at 30% (14/43), followed by social interaction or social encounter at 12% (5/43), and interaction quality or interpersonal relationship quality at 9% (4/43). The structural dimension of social connectedness (ie, social accompaniment and social interaction) was related to the functional dimension (ie, loneliness) [27,28,46,48-50,54,56,59] and quality dimension (ie, interaction quality) [34,52]. In addition, loneliness was associated with various factors such as individual factors (ie, age, gender, race, and ethnicity) [48,50,57], biological markers related to inflammation [45], psychosocial factors (ie, need to belong, positive and negative affect, and anxiety) [28,44,60], and contextual factors such as location [48,49]. Social interaction was associated with individual personality (ie, daily extraversion and neuroticism) [41] and well-being indicators (ie, mild cognitive impairment and fatigue) [47,55].

The eligibility criteria common to all studies was community-dwelling older adults. Based on the original data criteria, study-specific criteria included that eligible participants were not institutionalized [15,41-47], not working full-time [26-40], or involved in a prosocial program [61]. In terms of physical function or disease, exclusion criteria included visual or auditory impairments [15,41-47,52-56,62], active psychiatric symptomatology [15,41-47], cognitive impairment or diagnosis of dementia [15,41-47,58-60,62], nonambulatory status [15,41-47], and disabilities in activities of daily living [32] (Multimedia Appendix 4 [15,26-75]).

In terms of quality, according to the MMAT ratings, most studies met 100% of the criteria (*****, n=30), 8 studies achieved 80% (****), and 5 studies met 60% (***) of the criteria (Multimedia Appendix 5).

### EMA of Social Connectedness and Other Contextual Variables

As illustrated in Multimedia Appendix 6, most studies (38/43, 88%), except for 5 studies [40,44,45,57,63], focused on the structural dimension of social connectedness. These studies investigated whether an individual was engaged in social contact or interaction at a specific time. Furthermore, 17 studies (17/43, 40%) addressed the functional dimension, all of which addressed loneliness [27-29,43-46,48-50,54,56,57,59,60,63] except for 1 study that addressed social exchange [40]. The quality dimension covered in 16 studies (16/43, 37%) included assessments of the quality of social interactions [15,42,46,47,52,58,64], pleasantness of encounters or positive encounters [31,33,34,38,56,65], interpersonal tension [35], stress or negative social encounters [26,37,65], and stressful discussions [31,34,38]. Furthermore, 2 studies [46,56] explored all 3 dimensions of social connectedness.

Each dimension of the multifactorial construct of social connectedness was assessed using 1 or 2 questions. Several studies required participants to enumerate their social partners during the initial interview, specifying whether they had been in contact with any of these partners in the preceding 3 hours. Subsequently, these studies tracked whether participants had contact with the listed social partners at each assessed moment [26-40]. While studies examining social relationships have considered a wider range of interaction types, such as in-person, phone, computer, or text [15,26-28,30,33,34,39,41,47-56, 58,60-62,65], some studies have focused on in-person [48-51,62].

Other variables measured by EMA included context, such as location [32,43,48-51,53,66], activity or behavior [28,32,34,36,46,47,51,61,63], sedentary time [36], and time spent exercising and outdoors [60], and cognitive function [15,43]. In addition, concurrent positive or negative moods [26,29-31,34-40,44,46,54,56,57,59-63,67], pain [33,37,51,57], fatigue [51,57], stress [41,42,51], and sense of relatedness [52,61] were also assessed.

### Social Connectedness at Trait-Level Assessment

Most studies (40/43, 93%) addressed at least 1 dimension of social connectedness at trait-level assessment: the structural dimension, such as marital status, living arrangement, or social network (40/43, 93%); the functional dimension, such as loneliness or social support (6/43, 14%); and the quality dimension, such as negative social interaction and social strain (4/43, 9%). Each study used this information to describe the sample characteristics at trait-level assessment, to compare outcomes measured by EMA, or to include them as control variables in the analysis (Multimedia Appendix 7).

### EMA Protocols

Multimedia Appendix 8 [15,26-75] summarizes details of EMA varied across data sources. Among the 15 data sources, the design of EMA prompts was categorized as follows: 6 data sources employed a quasi-random scheduling method for delivering prompts [15,26-51,58,60,62], while 4 data sources used a self-determined approach in which participants responded at a time of their choosing before bedtime [57,59,63,65]. Furthermore, 2 data sources used a completely randomized schedule [61,66]. Event-contingent designs, in which specific events triggered prompts [52-56] and fixed time [64] were each in 1 data source, respectively. In addition, 1 data source did not specify the prompt design method [61].

The frequency of survey administration ranged from 1 to 7 times daily, with the assessment periods lasting between 5 and 25 days. Most data sources reported surveys via smartphones, although 6 data sources did not specify the device type used [57,61,62,65-67]. Digital devices were used to capture voice data to estimate conversation frequency [30] and to collect accelerometer data to assess physical activity [36]. All but 9 data sources [52-57,59-61,63,64,66,67] described details regarding participant training before commencing actual data collection. Depending on participant responses, reminders were issued in a predetermined manner [48-51,57,59,60,63]. In addition, 1 data source took proactive measures by contacting participants if they missed 3 consecutive surveys, to address any technical or adherence problems [60]. Data were included in the analysis if they were submitted within a pre-established maximum time frame.

### Definition and Rate of Compliance and Adherence to EMA

As illustrated in Multimedia Appendix 9, studies used the terms completion, compliance, and adherence. In 1 study [49], adherence was defined as the number of valid EMAs divided by the potential maximum EMA number. The completion rate for studies in the overall adult population was 89% [58], while those targeting only older adults—based on 2 data sources—reported completion rates of 70% or higher [28,33,61]. Among the older adult population, based on 5 data sources, compliance with EMA protocol instructions exceeded 82% [42,44,46,47,54,56,57,63,66]. Although some studies used the same data source, the number of valid EMAs varied depending on the specific aims of each study, resulting in differences in compliance rates [42,44,46,47]. Only 1 study presented a predefined acceptable adherence rate of at least 75%, with a 7-day adherence rate of 83.9% [60]. In contrast, a study using the Chicago Health and Activity Space in Real-Time data with a 6-wave design had an adherence rate of 58% [49]. In studies that combined beep and end-of-day surveys, end-of-day surveys showed lower levels of compliance compared to beep surveys [42,44,46,47], whereas it was higher in the evening compared with the morning or afternoon [60].

Few studies have specifically identified reasons for participants’ lack of complete EMA evaluations. Among those that did, technical issues with EMA [32,50,51] and misunderstandings of the instructions [52,55] were commonly reported. Although respondents received direct training, including on how to use devices, 15% (72/455) of respondents were unable to complete the EMA due to unfamiliarity with using smartphones [51].

### Conceptual Summary of Social Connectedness

As illustrated in Table 1, we summarized how the selected studies addressed the 3 dimensions of social connectedness in their EMA assessments, using the framework proposed by Holt-Lunstad et al [8]. The table distinguishes between trait-level characteristics and dynamic, real-time measures captured through EMA, which include both momentary snapshots and daily summaries. It also highlights the role of contextual factors in enhancing the understanding of social connectedness. Specifically, EMA captures the structural dimension through indicators such as the occurrence, frequency, characteristics of social partners, and the purpose of interactions; the functional dimension through measures of loneliness and the exchange of emotional or instrumental support; and the quality dimension through the subjective evaluation of interactions, including pleasantness, stress, and interpersonal tension. While many indicators are measured consistently across time units, certain aspects (eg, stressful discussions) were assessed only at specific moments due to the limitations of self-reporting or observation within short time frames. Overall, these findings underscore social connectedness in later life as a dynamic construct shaped by the interplay of structure, function, and quality across varying contexts and temporal scales.

**Table 1:** Conceptual summary of social connectedness.

Dimension	Trait-level assessment	Ecological momentary assessment				Context
		Momentary (right now)	Since the last assessment	Today as a whole		
Structure	Living arrangement Marital status Household composition Extent and frequency of social connection Social network size Proportion time spent alone Social interaction average frequency Leadership role in prosocial activity The number of close social relationships The overall contact frequency with relationship partners	Social interaction Occurrence Duration (interaction time) Social partner characteristics Modality Interaction purpose	Social interaction Occurrence Frequency Social partner characteristics Modality	Social interaction Occurrence Duration (time spent alone)	Behavioral context Engagement in daily activity Physical activity and sedentary time^a^ Outdoor activity and exercise time Social media use Functional context Functional ability Cognitive function Environmental context Location	
Function	Loneliness Social support Emotional, instrumental, or informational support	Loneliness	Loneliness	Loneliness Provision and receipt of support and advice		
Quality	Social interaction quality Social strain Negative social interaction Social network quality	Social interaction quality Pleasant Valence, satisfaction, feeling	Stressful discussion Interpersonal tensions Social interaction quality Pleasant, unpleasant, or both or neutral Positive or negative	Social interaction quality Positive or negative		

^a^Indicators using accelerometer assessment.

## Discussion

### Principal Findings

In this integrative review, we identified and evaluated studies that assessed social connectedness in older adults using EMA. We examined how EMA was used to assess the multifactorial construct of social connectedness and gather contextual information. In addition, we highlighted the procedural elements of EMA identified in each study. Overall, using EMA to measure social connectedness in community-dwelling older adults is considered feasible, and refining these procedural elements will enhance the utility of EMA for this demographic. With the rapid advancement of digital technologies and an increasing emphasis on data collection methods that are more centered around participants’ needs and preferences, EMA methods are expected to become more personalized and user-friendly. This review is particularly timely as it is among the first to comprehensively evaluate the use of EMA in measuring social connectedness specifically within the older adult population. By examining the current state of EMA methods and identifying gaps in these approaches, this review provides valuable insights that can guide future research in this area.

In our review, the structural dimension of social connectedness, such as social accompaniment or interaction, was most frequently examined in EMA studies. Only 2 studies [46,56] addressed all 3 dimensions—structural, functional, and quality—while the others focused on just 1 or 2 dimensions. A multifactorial approach to social connectedness emerged as the strongest predictor of mortality risk, comparable in magnitude with well-recognized health determinants like alcohol consumption and smoking [76]. In addition, this multifactorial approach aids in the appropriate allocation of limited resources for social support interventions [8,77]. Despite the benefits of a multifactorial approach, previous studies have often focused on limited dimensions of social connectedness. Many of the studies included in this review were secondary analyses based on the same dataset, which may have resulted in limited variable selection and a lack of diversity in variables. Future research should consider adopting a multifactorial approach to achieve a more nuanced understanding of social connectedness, thereby enabling a more comprehensive reflection and interpretation of its components.

Across the 43 studies reviewed, the EMA protocols demonstrated consistency in methodology, largely because most studies adopted established protocols from large-scale projects. These protocols outlined the optimal frequency, duration, and intervals for data collection, as well as the devices used. They also featured carefully selected questions designed to boost participant response rates and enhance data accuracy, with 1-2 items being used intensively in EMA. Although there is increasing evidence suggesting that fewer items may still provide adequate validity [78,79], concerns remain about whether these items sufficiently capture the study’s target construct and provide enough information. Internal consistency (eg, Cronbach α) often increases as the number of items increases [80]. Yet, a previous study pointed out that an increased number of items can also increase participant burden and compromise the data quantity and quality in the experience sampling method [81]. Therefore, when selecting items for EMA, balancing internal consistency and participant burden is essential for effectively assessing social connectedness through EMA in older adults. For aspects that cannot be measured in real time, such as social network composition, supplementing EMA with methods like trait-level assessment could be a viable alternative.

Adopting individualized protocols and collecting real-time information were key to ensuring ecological validity. Most studies aimed to accommodate participants’ circumstances better and minimize disruption to their daily routines. In addition, gathering real-time data on participants’ locations [32,43,48-51,53,66], physical or social contexts [28,32,34,36, 46,47,51,60,61,63], cognitive function [15,43], physical symptoms [33,37,51,57], and mood [26,29-31,34-40, 44,46,54,56,57,59-63,67] played a crucial role in understanding the contexts of social connectedness among older adults. However, it is noteworthy that while the studies defined the types of social interactions, they reported the results without specifying the mode of social interaction, such as in-person versus technology-mediated interactions. Some studies specifically focused on in-person interactions, considering technology-mediated interactions either irrelevant to well-being [54] or representing a minor portion of the data (eg, 8% [1198/15,479 encounters] text messaging) [30]. Smartphone ownership rates have been increasing globally, particularly among US adults aged 65 years and older, rising from 13% in 2012 to 61% in 2021. Similarly, internet usage among older adults has followed a comparable trend [82]. In particular, older adults’ technology-based social experiences have increased since the COVID-19 pandemic, and are likely to differ compared with prepandemic times [83], which suggests that this may have led to changes in social interaction modality and relationships. Given that most studies were conducted before the pandemic or during the pandemic, future research designs should carefully consider individual circumstances and the changing social landscape to provide a nuanced understanding of real-time social connectedness in the older adult population.

Most studies have relied on self-reported outcomes from older participants using digital devices. Real-time data collection through these devices may face challenges due to technical issues (eg, malfunctions), physical limitations (eg, hearing impairment), and cognitive barriers (eg, forgetfulness) [84]. Given the steady aging of the global population, the potential for physical or cognitive impairments in older adults cannot be ignored [85,86]. Furthermore, data from self-report surveys might be skewed by participants’ concerns about the stigma associated with social isolation or loneliness [87], which can hinder the identification of individuals with low social connectedness. As a promising alternative, unobtrusive passive sensing technologies such as GPS, accelerometers, light sensors, phone usage, voice monitors, and vital signs can minimize the need for active participant involvement in specific situational settings, potentially circumventing many data collection issues [88,89]. As an example, mobile data of phone activity (eg, SMS text messages and call logs) was used to infer communication (ie, social interactions) [90]. In addition, passive data have been used to predict loneliness [91-93], for example, objective physiological parameters (ie, heart rate, heart rate variability, physical activity, and sleep) collected with smartwatches to build a model to predict loneliness [93]. These prediction algorithms could be further developed to enhance support programs. Some studies included in the review showed the potential to integrate passive data from voice recordings [30] or wearable devices to measure social connectedness [36]. The combination of various passive metrics in existing approaches is expected to yield complementary insights [88,89]. However, passive measures have limitations in fully capturing subjective dimensions (ie, functional and quality dimensions of social connectedness) [94,95], therefore, it is necessary to use active and passive data complementarily.

The results of this review highlight important considerations in future research design. First, our analysis showed that social connectedness is not merely the presence or frequency of encounters but involves unidirectional or bidirectional relationships across structural, functional, and quality dimensions. These dimensions were closely related to personal [48,50,57], biological [45], psychosocial [28,44,60], and contextual factors [48,49]. Identifying additional contextual factors is crucial to understanding social connectedness and pinpointing those at risk, thereby guiding the development of effective intervention strategies. By more precisely assessing factors that require intervention, tailored support can be provided to enhance social connectedness in older adults. However, current research often overlooks the multifaceted nature of social connectedness, especially its structural, functional, and quality dimensions. Furthermore, social connectedness measures for older adults are often modeled after those for younger adults, which may not account for unique factors like retirement [96,97]. Therefore, a comprehensive understanding of social connectedness is necessary, with careful attention to selecting relevant variables to better understand the experiences of older adults.

Second, the older adult population showed relatively high EMA compliance and adherence rates. Some studies have also shown that older adults have higher reporting fidelity and accuracy than young adults [13,98]. Therefore, EMA studies targeting older adults are likely to secure reliable data. However, technical challenges remain in conducting EMA using digital devices, suggesting that older adults’ digital access and familiarity with devices may affect compliance [48-51]. Although not explicitly discussed in the included studies, it is possible that discomfort with unfamiliar digital devices may negatively affect EMA compliance [99]. Therefore, it is important to improve older adults’ digital accessibility and device usage skills and improve research methods. Typically, the training session is used to familiarize participants with how to use the device. However, incorporating hands-on training and simple EMA simulations (eg, 1- to 2-day mock surveys) in addition to basic training is considered a more effective approach. In addition, when analyzing the changes in EMA compliance according to older adults’ daily schedule, the “end-of-day” survey showed lower compliance than the “beep survey” [42,44,46,47]. These results are consistent with those of previous studies reporting significantly lower survey response rates in the evening than in the morning or afternoon [100]. Meanwhile, 1 study showed that the response rate in the night survey was lower than the morning and evening surveys [60]. The variation across studies suggests that temporal variations in motivation, activity level, context, or individual factors may have influenced older adults’ willingness and opportunity to respond to EMA [100]. Therefore, future EMA studies should investigate participants’ experiences after the end of the study to better understand the context of response patterns. This will be an important step in establishing the EMA methodology as feasible and effective beyond understanding the social connectedness.

### Limitations

This study has several limitations. First, although we included 5 databases, we did not include all possible databases, which may have resulted in missing some studies that met the inclusion criteria. Second, most of the studies used identical datasets, thus limiting the diversity of protocols available for analysis. Nevertheless, the provision of well-structured protocols in cohort studies with large sample sizes offers valuable insights, which are beneficial for designing future EMA studies in older adults. Finally, the study sample consisted of older adults who exhibit relatively good physical and cognitive functioning, which limits the generalizability of the findings. Therefore, the findings should be interpreted with caution. Future EMA studies should aim to explore the feasibility of EMA in more diverse and heterogeneous groups of the older adult population, such as those with lower levels of physical and cognitive functioning.

### Conclusions

This study highlights the need for both conceptual and methodological refinements in using EMA to measure social connectedness among older adults. It is necessary to capture the multifactorial construct of social connectedness in real time, and the unique experience of EMA, particularly with digital devices, should be incorporated into the design process. Further research with diverse older adult populations is recommended to better understand and address barriers to adherence to EMA protocols for measuring social connectedness.

## Appendix

Multimedia Appendix 1 PRISMA (Preferred Reporting Items for Systematic reviews and Meta-Analyses) checklist.

Multimedia Appendix 2 Search strategies.

Multimedia Appendix 3 Summary of included studies (n=43).

Multimedia Appendix 4 Inclusion and exclusion criteria by data source.

Multimedia Appendix 5 Quality evaluation of the selected studies.

Multimedia Appendix 6 Ecological momentary assessment of social connectedness and other contextual variables.

Multimedia Appendix 7 Trait-level assessment of social connectedness.

Multimedia Appendix 8 Ecological momentary assessment protocols.

Multimedia Appendix 9 Definition and rate of compliance and adherence used in selected studies.

## Abbreviations

CHART: Chicago Health and Activity Space in Real-Time
EMA: ecological momentary assessment
MeSH: Medical Subject Headings
MMAT: Mixed-Methods Appraisal Tool
PRISMA: Preferred Reporting Items for Systematic Reviews and Meta-Analyses
