# Supplementary material for: Ecological Momentary Assessment to Measure Social Connectedness in Older Adults: Integrative Review
Source: J Med Internet Res. 2025 Jun 17;27:e66324. doi: 10.2196/66324 (PMC12214698; doi:10.2196/66324)
Supplement: Multimedia Appendix 2 [file jmir_v27i1e66324_app2.docx]

# Multimedia Appendix 2. Search strategies

| Database | | Query | Result |
| --- | --- | --- | --- |
|  | |  |  |
| **PubMed** | |  |  |
|  | #1 | ("Aged"[Mesh]) OR (older OR older adults OR older people OR senior OR seniors OR old age OR old person OR elderly OR elder OR elders OR aging adult OR aging adults OR ageing adult OR ageing adults OR aged) | 7,230,165 |
|  | #2 | ("Ecological Momentary Assessment"[Mesh]) OR ("ecological momentary assessment" OR "experience sampling" OR "ecological momentary" OR "momentary assessment" OR "ambulatory assessment" OR "event sampling" OR "real time assessment" OR "real time data" OR ("daily life" AND (report* OR assessment*)) OR "daily diar*" OR "intensive longitudinal data" OR "intensive longitudinal methods" OR "intensive longitudinal assessment" OR "intensive longitudinal research" OR "intensive longitudinal stud*") | 29,959 |
|  | #3 | (((((("Interpersonal Relations"[Mesh]) OR ("Loneliness"[Mesh])) OR ("Social Isolation"[Mesh])) OR ("Social Interaction"[Mesh])) OR ("Social Support"[Mesh])) OR ("Social Networking"[Mesh])) OR (interpersonal relations OR social relationship* OR loneliness OR social isolation OR social exclusion OR perceived isolation OR social interaction OR social support OR social networking OR social connection OR social connectedness) | 1,108,900 |
|  |  | #1 AND#2 AND #3 | 2,447 |
| **CINAHL** | |  |  |
|  | #1 | (MH "Aged+") OR ((older) OR (older adults) OR (older people) OR (senior) OR (seniors) OR (old age) OR (old person) OR (elderly) OR (elder) OR (elders) OR (aging adult) OR (aging adults) OR (ageing adult) OR (ageing adults) OR (aged)) | 1,292,291 |
|  | #2 | ("ecological momentary assessment") OR ("experience sampling") OR ("ecological momentary") OR ("momentary assessment") OR ("ambulatory assessment") OR ("event sampling") OR ("real time assessment") OR ("real time data") OR (("daily life" AND (report* OR assessment*))) OR ("daily diar*") OR ("intensive longitudinal data") OR ("intensive longitudinal methods") OR ("intensive longitudinal assessment") OR ("intensive longitudinal research") OR ("intensive longitudinal stud*") | 11,131 |
|  | #3 | ((MH "Interpersonal Relations+") OR (MH "Loneliness") OR (MH "Social Isolation+") OR (MH "Support, Social+") OR (MH "Social Networking+")) OR ((interpersonal relations) OR (social relationship*) OR (loneliness) OR (social isolation) OR (social exclusion) OR (perceived isolation) OR (social interaction) OR (social support) OR (social networking) OR (social connection) OR (social connectedness)) | 511,393 |
|  |  | #1 AND#2 AND #3 | 529 |
| **Embase** | |  |  |
|  | #1 | ('aged'/exp) OR (older) OR (older AND ('adults'/exp OR adults)) OR (older AND people) OR (senior) OR (seniors) OR (old AND ('age'/exp OR age)) OR (old AND person) OR ('elderly'/exp OR elderly) OR (elder) OR (elders) OR (('aging'/exp OR aging) AND ('adult'/exp OR adult)) OR (('aging'/exp OR aging) AND ('adults'/exp OR adults)) OR (('ageing'/exp OR ageing) AND ('adult'/exp OR adult)) OR (('ageing'/exp OR ageing) AND ('adults'/exp OR adults)) OR ('aged'/exp OR aged) | 7,386,348 |
|  | #2 | ('ecological momentary assessment'/exp) OR ('ecological momentary assessment'/exp OR 'ecological momentary assessment') Or ('experience sampling'/exp OR 'experience sampling') OR ('ecological momentary') OR ('momentary assessment') OR ('ambulatory assessment'/exp OR 'ambulatory assessment') OR ('event sampling') OR ('real time assessment') OR ('real time data') OR (('daily life'/exp OR 'daily life') AND ((report*) OR (assessment*)) OR ('daily diar*') OR ('intensive longitudinal data') OR ('intensive longitudinal methods') OR ('intensive longitudinal assessment') OR ('intensive longitudinal research') OR ('intensive longitudinal stud*') | 111,288 |
|  | #3 | (('social connectedness'/exp) OR ('human relation'/exp) OR ('loneliness'/exp) OR ('social isolation'/exp) OR ('social interaction'/exp) OR ('social support'/exp) OR ('social network'/exp)) OR ((interpersonal AND relations) OR (('social'/exp OR social) AND relationship*) OR ('loneliness'/exp OR loneliness) OR (('social'/exp OR social) AND ('isolation'/exp OR isolation)) OR (('social'/exp OR social) AND exclusion) OR (perceived AND ('isolation'/exp OR isolation)) OR (('social'/exp OR social) AND ('interaction'/exp OR interaction)) OR (('social'/exp OR social) AND ('support'/exp OR support)) OR (('social'/exp OR social) AND networking) OR (('social'/exp OR social) AND ('connection'/exp OR connection)) OR (('social'/exp OR social) AND ('connectedness'/exp OR connectedness))) | 1,742,448 |
|  |  | #1 AND#2 AND #3 | 12,162 |
| **Web of Science** | |  |  |
|  | #1 | ALL=(older OR older adults OR older people OR senior OR seniors OR old age OR old person OR elderly OR elder OR elders OR aging adult OR aging adults OR ageing adult OR ageing adults OR aged) | 6,102,769 |
|  | #2 | ALL=("ecological momentary assessment" OR "experience sampling" OR "ecological momentary" OR "momentary assessment" OR "ambulatory assessment" OR "event sampling" OR "real time assessment" OR "real time data" OR ("daily life" AND (report* OR assessment*)) OR "daily diar*" OR "intensive longitudinal data" OR "intensive longitudinal methods" OR "intensive longitudinal assessment" OR "intensive longitudinal research" OR "intensive longitudinal stud*") | 55,351 |
|  | #3 | ALL=(interpersonal relations OR social relationship* OR loneliness OR social isolation OR social exclusion OR perceived isolation OR social interaction OR social support OR social networking OR social connection OR social connectedness)" | 1,525,374 |
|  |  | #1 AND#2 AND #3 | 2,253 |
| **PsycINFO** | |  |  |
|  | #1 | MAINSUBJECT.EXACT.EXPLODE("Aged (Attitudes Toward)") OR older OR older adults OR older people OR senior OR seniors OR old age OR old person OR elderly OR elder OR elders OR aging adult OR aging adults OR ageing adult OR ageing adults OR aged | 955,314 |
|  | #2 | MAINSUBJECT.EXACT.EXPLODE("Ecological Momentary Assessment") OR "ecological momentary assessment" OR "experience sampling" OR "ecological momentary" OR "momentary assessment" OR "ambulatory assessment" OR "event sampling" OR "real time assessment" OR "real time data" OR ("daily life" AND (report* OR assessment*)) OR "daily diar*" OR "intensive longitudinal data" OR "intensive longitudinal methods" OR "intensive longitudinal assessment" OR "intensive longitudinal research" OR "intensive longitudinal stud*" | 18,397 |
|  | #3 | MAINSUBJECT.EXACT.EXPLODE("Social Connectedness") OR MAINSUBJECT.EXACT.EXPLODE("Interpersonal Relationships") OR MAINSUBJECT.EXACT.EXPLODE("Loneliness") OR MAINSUBJECT.EXACT.EXPLODE("Social Isolation") OR MAINSUBJECT.EXACT.EXPLODE("Social Interaction") OR MAINSUBJECT.EXACT.EXPLODE("Social Support") OR interpersonal relations OR social relationship* OR loneliness OR social isolation OR social exclusion OR perceived isolation OR social interaction OR social support OR social networking OR social connection OR social connectedness | 1,524,412 |
|  |  | #1 AND#2 AND #3 | 1,495 |
