# Supplementary material for: Ecological Momentary Assessment to Measure Social Connectedness in Older Adults: Integrative Review
Source: J Med Internet Res. 2025 Jun 17;27:e66324. doi: 10.2196/66324 (PMC12214698; doi:10.2196/66324)
Supplement: Multimedia Appendix 3 [file jmir_v27i1e66324_app3.docx]

Multimedia Appendix 3. Summary of included studies (n=43)

| First author, year / Country | Purpose | Sample | | Data source | Main finding | Factors associated with social connectedness  (Outcome) | MMAT^b^  quality appraisal  (Max *****) |
| --- | --- | --- | --- | --- | --- | --- | --- |
|  |  | Sample size, n (Female, %) | Age, year (Mean±SD^a^) |  |  |  |  |
|  |  |  |  |  |  |  |  |
| Compernolle EL, 2024 / USA [48] | To examine how momentary loneliness relates to gender and race/ethnicity and social and physical context | 342  (60.0%) | 73.69 | CHART | • Older adults were less likely to feel lonely when outside their homes or not alone, although this effect was weaker for women, Black, and Hispanic older adults. | (Loneliness)  • Momentary social accompaniment and context (i.e., location)  • Gender^a^  • Race and ethnicity^a^ | ***** |
| Compernolle EL, 2024 / USA [49] | To examine real-time contextual influences on momentary loneliness in older adults compared to before the COVID-19 crisis | 110  (51.8%) | 72.35±5.54 | CHART | • Older adults experienced more intense momentary loneliness during the pandemic than before but were significantly less lonely when at home and/or alone. | (Loneliness)  • Momentary social accompaniment and context (i.e., location) | ***** |
| Ferguson G, 2024 / USA [41] | To investigate how daily and trait measures of extraversion and neuroticism related to daily occurrences of stressors and social interactions | 110  (65.2%) | 79.21±5.52 | EAS | • Daily extraversion and daily neuroticism positively predicted social interaction, while stressors were negatively predicted by daily extraversion and positively predicted by daily neuroticism. As a global measure, trait neuroticism showed a significantly positive relationship with stressors. | (social interaction)  • Daily extraversion  • Daily neuroticism | ***** |
| Fingerman KL, 2024 / USA [26] | To explore the encounters with each type of tie associated with older adults’ interpersonal stress and mood and whether believing that one has many long-duration relationships moderates social stress and mood | 313  (56.0%) | 73.94±6.38 | DEWS | • Long-duration ties with infrequent contact exacerbated the effects of interpersonal stress on mood.  • Active ties that involved regular and frequent contact helped buffer interpersonal stress regardless of duration of the ties, while long-duration ties without contact worsened its impact. | • Not applicable | ***** |
| Hülür G, 2024 / Switzerland [52] | To understand differences in the perceived quality of daily social interactions across interaction modalities and to examine whether these differences are moderated by the purpose of the social interaction. | 118  (40.0%) | 72±5 | Specific name not mentioned^b^ | • Text-based communication was rated lower in valence and social relatedness relative to face-to-face communication and telephone calls, and lower in meaningfulness relative to telephone calls. Face-to-face and telephone communication differed in meaningfulness, with telephone calls rated higher.  • Some of the associations between interaction modality and perceived quality were moderated by interaction purpose. | (Interaction quality)  • Interaction modality  • Interaction purpose | ***** |
| Jang H, 2024 / USA [42] | To investigate the mediating role of daily psychological stress in the relationship between the quality of daily social encounters and cognitive decline in older adults | 254  (67.7%) | 76.5 | EAS | • Ambivalent and neutral social encounters were positively associated with daily stress levels. Between-person daily stress was linked to increased subjective cognitive decline.  • There was a significant indirect path from ambivalent social encounters to SCD through daily stress. | • Not applicable | ***** |
| Kang JE, 2024 / USA [43] | To examine the association between within-person variation in state loneliness and cognitive performance assessed objectively in daily life | 313  (67.5%) | 77.47±4.85 | EAS | • Momentary assessments of increased loneliness were consistently linked to worse cognitive performance on concurrent assessments. Moments characterized by lower cognitive performance predicted higher levels of loneliness 3–4 hours later (next occasion), but not vice versa. | • Not applicable | ***** |
| Luo MX, 2024 / Switzerland [53] | To examine routineness of social interactions in relation to cognition and well-being in community-dwelling older adults’ daily life | 103  (40.0%) | 71.4±4.4 | Specific name not mentioned^b^ | • The higher routineness of social interaction was associated with higher positive and lower negative affect. Social interaction in the same modality and with the same partner type increased positive affect. However, routineness of social interactions was not associated with life satisfaction or cognitive abilities. | • Not applicable | ***** |
| Wallimann M, 2024 / Switzerland [59] | To investigate associations of daily solitude with subjective and physical well-being under consideration of time-savoring | 108  (58.0%) | 73.11±5.93 | Not applicable | • Everyday solitude was associated with higher depressive mood and loneliness among older individuals. Everyday time-savoring was related to lower depressive mood, loneliness, and somatic symptoms.  • Daily time-savoring attenuated the association of daily solitude with depressive mood, loneliness, and somatic symptoms. | (Loneliness)  • Daily solitude  • Daily time-saving | ***** |
| Zhang S, 2024 / USA [27] | To explore different modes of social contact associated with older adults’ loneliness, and whether such links differ by the type of social ties | 313  (56.0%) | 73.94±6.38 | DEWS | • Momentary loneliness predicted a greater likelihood of phone contact in the next 3 hours. However, only in-person contact was associated with lower levels of loneliness.  • Momentary loneliness was associated with more in-person and phone contact with close ties, yet fewer in-person contacts with weak ties. In-person contact with both close and weak ties predicted lower levels of loneliness. | (Loneliness)  • Social contact mode with social tie | ***** |
| Zhou ZX, 2023 / USA [28] | To examine the associations between older adults’ need to belong, daily social engagement, and transient loneliness in their everyday life | 299  (55.0%) | 73.71±6.25 | DEWS | • Older adults with a higher need to belong are more likely to feel lonely, but this is attenuated by in-person encounters with a greater variety of social partners and group engagement throughout the day. | (Loneliness)  • Need to belong^a^  • Daily social engagement (types of social partners encountered, mode of contact) | ***** |
| Goldman AW, 2023 / USA [50] | To examine how personal network size relates to the association between social accompaniment and loneliness in a real-time framework | 343  (60.0%) | 73.61±6.60 | CHART | • Older adults with large personal networks experienced more intense momentary loneliness than those with smaller social networks when momentarily alone, particularly notable among men. | (Loneliness)  • Social network size according to momentary social accompaniment  • Gender^a^ | **** |
| Goldman AW, 2023 / USA [51] | To examine how being with social accompaniment is associated with momentary symptoms | 342  (62.0%) | 73.78±6.58 | CHART | • Older adults who were physically in the company of a friend or neighbor were significantly less likely to experience momentary fatigue and stress. | • Not applicable | **** |
| Van Bogart, 2023 / USA [44] | To examine bidirectional associations between loneliness and anxiety during daily life | 317  (67.5%) | 77.45±4.83 | EAS | • Significant bidirectional associations between loneliness and anxiety were present contemporaneously and 3–4 hours later, but higher momentary loneliness only predicted higher end-of-day anxiety. Higher average daily loneliness predicted higher end-of-day anxiety, but not the vice versa. | (Loneliness)  • Anxiety | ***** |
| Badal VD, 2022 / USA [60] | To investigate sequential association between momentary loneliness, affect, and social behavior | 22  (86.4%) | 80.24±7.13 | CCSHC study's pool | • Loneliness was contemporaneously associated with negative affect. Negative affect tended to be followed by loneliness and then by exercise or outdoor physical activity. | (Loneliness)  • Positive and negative affect | *** |
| Kim YK, 2022 / USA [29] | To investigate the associations between daily social media use, daily emotional well-being, daily social encounters, and social network in later life | 310  (56.0%) | 73.95±6.39 | DEWS | • Individuals experienced less negative mood on days with more social media use and more in-person encounters.  • More daily social media use was associated with more positive mood for individuals with a relatively small social network but not for their counterparts with larger social networks. | • Not applicable | ***** |
| Luo M, 2022 /Switzerland   [54] | To examine diminishing returns to social interaction frequency for well-being | 116  (41.0%) | 71.77±5.10 | Specific name not mentioned^b^ | • A higher number of daily social interactions was associated with higher daily positive affect and lower daily negative affect and loneliness. But well-being peaked at 2–3 times above typical daily interaction frequency, and the benefits diminished beyond that point. | (Loneliness)  • Social interactions (frequency) | ***** |
| Luo M, 2022 / Switzerland [55] | To examine alternation patterns of social interactions and solitude in healthy and community- dwelling older adults | 118  (40.0%) | 72±5 | Specific name not mentioned^b^ | • A longer-than-usual social interaction preceded and was followed by a longer-than-usual solitude episode, and vice versa. These processes were in part moderated by older adults' trait life satisfaction and trait fatigue, but only for the solitude-then-interaction association. | (Social interaction)  • Solitude  • Trait fatigue^a^  • Life satisfaction^a^ | ***** |
| Mann AS, 2022 / USA [61] | To investigate whether pursuing formal prosocial activity in solitude predicts higher levels of well-being compared to pursuing other activities when alone | 165  (59.9%) | 71.13±5.70 | Specific name not mentioned^c^ | • Positive affect increased during prosocial-program activity, whether alone or with others, while negative affect rose only in the presence of others. Older adults felt more relatedness during prosocial-program activity in solitude compared to other solitary activities. | • Not applicable | **** |
| Ng YT, 2022 / USA [30] | To understand the relationship between frequency of conversation and mood throughout the day according to marital status in older adults | 272  (54.0%) | 74.01±6.36 | DEWS | • Married older adults had more conversations than divorced ones, while divorced individuals engaged more with friends throughout the day. Conversations positively affected mood, particularly noticeable among widowed individuals compared to married ones. | (Conversations and social encounters)  • Marital status^a^ | ***** |
| Pfund GN, 2022 / Switzerland [64] | To explore whether the quality of daily social interactions is associated with the level of daily purpose in older adults and the covariates that modify this association | 104  (51.9%) | 70.75±7.23 | MUAWO | • When individuals had more positive social interactions, they felt more purposeful. Employment status moderated this association with stronger association in unemployed/retired individuals. | • Not applicable | *** |
| Van Bogart K, 2021 / USA [45] | To examine the associations between loneliness (trait and momentary) and both basal and stimulated inflammatory marker among older adults | 222  (63.0%) | 76.82±4.72 | EAS | • Both higher trait loneliness and aggregated momentary measures of loneliness were positively associated with CRP levels. However, there were no significant associations between loneliness and basal or stimulated cytokines. | (Momentary loneliness and trait loneliness)  • Inflammation (CRP; beginning and end of 14-day) | **** |
| Zhang S, 2022 / USA [31] | To examine how daily social experiences and mood varied by narcissism among older adults | 304  (55.0%) | 73.85±6.32 | DEWS | • Narcissism did not predict the number or pleasantness of social encounters. More narcissistic individuals may be less sensitive to a close partner’s distress, but their moods may be more susceptible to negative social events, especially with weak ties. | • Not applicable | ***** |
| Zhaoyang R, 2022 / USA [46] | To examine the dynamic associations between social interactions and momentary loneliness in older adults' daily lives, and the influence of individual differences in trait loneliness and neuroticism on these dynamics | 317  (67.5%) | 77.45±4.83 | EAS | • Having more frequent, more pleasant, and in-person social interactions, as well as interactions with family and friends were bidirectionally associated with momentary loneliness. Trait loneliness and neuroticism moderated the within-person effects of social interactions on momentary loneliness. | (Loneliness)  • Social interactions  • Trait loneliness and neuroticism^a^ | ***** |
| Fingerman KL, 2021 / USA [32] | To investigate association with functional limitations and activities throughout the day in older adults and the moderating role of social integration on this relationship | 313  (56.0%) | 73.94±6.38 | DEWS | • Functional limitations were associated with an increased likelihood of activities of TV watching and medical appointments.  • Individuals with functional limitations were more likely to attend medical appointments when with their social partners than when alone. | • Not applicable | ***** |
| Huo M, 2021 / USA [33] | To examine the bidirectional association between older adults' pain and nighttime sleep disturbances and the moderating role of daily positive encounters in these associations | 292  (55.0%) | 73.71±6.28 | DEWS | • More prior nighttime sleep disturbances were associated with more severe pain the next day. This association weakened on days when a greater proportion of positive or pleasant encounters were reported, especially with close partners. | • Not applicable | ***** |
| Junghaenel DU, 2021 / USA [57] | To examine age differences in the memory-experience gap for emotional, social, and physical well-being; and to examine factors associated with these age differences | 147^d^  (40.1%) | 70.68±4.36 | Not applicable | • Older adults, compared to young and middle-aged adults, had a smaller memory-experience gap for negative affect and loneliness. Lower day-to-day variability partially explained this smaller gap for older adults. | (Memory-experience gap for loneliness)  • Age^a^ | **** |
| Macdonald B, 2021 / Switzerland [56] | To examine associations between daily social interactions and daily well-being in older adults | 115  (40.0%) | 71.75±5.12 | Specific name not mentioned^b^ | • More daily face-to-face social interactions were associated with higher positive affect, and more digital interactions were associated with lower negative affect. On days with more face-to-face social interactions than average, higher positive affect, lower negative affect and loneliness were reported than usual. | (Loneliness)  • Social interaction | ***** |
| Ng YT, 2021 / USA [34] | To examine the emotional experiences when older adults encounter friends throughout the day | 313  (56.0%) | 73.94±6.38 | DEWS | • Encounters with friends were more pleasant and involved fewer stressful discussions than those with romantic partners or family. Friend encounters were also associated with better mood, particularly with less close friends. | (Quality of daily encounters)  • Social partners type | ***** |
| Zhaoyang R, 2021 / USA [15] | To examine the dynamic associations between daily social interactions and cognitive performance over different timescales | 312  (67.0%) | 76.97±4.85 | EAS | • Daily social interaction, especially having pleasant and frequent social interaction with close social relationships was a predictor of subsequent changes in daily cognitive function in older adults. | • Not applicable | **** |
| Zhaoyang R, 2021 / USA [47] | To examine whether features of daily social interactions serve to distinguish older adults with MCI from those without MCI | MCI:  100 (67.0%)  Non-MCI: 211 (67.4%) | MCI: 77.79±4.90  Non-MCI: 76.58±4.80 | EAS | • Older adults with MCI had less frequent total and positive social interactions and less frequent in-person socializing activities than those with normal cognitive function. However, there were no differences in conventional global measures. | (Social interactions)  • MCI^a^ | ***** |
| Birditt KS, 2020 / USA [35] | To examine links between interpersonal tensions and daily emotional well-being, and whether those links were moderated by general emotion regulation strategy preferences | 293  (54.9%) | 73.73±6.28 | DEWS | • Interpersonal tensions adversely affected emotional well-being, particularly among the oldest-old compared to younger-old individuals. Those who preferred reappraisal showed mitigated effects of tension, especially among those with poorer self-reported health. | (Daily interpersonal tensions)  • Preferences for emotion regulation strategies^a^ | ***** |
| Fingerman KL, 2020 / USA [36] | To examine the associations between social ties and diversity of daily behaviors, physical activity, non-sedentary time, and positive emotions | 313  (56.0%) | 73.73±6.28 | DEWS | • Encounters with a diverse range of social ties were linked to engaging in various behaviors, increased physical activity, and reduced sedentary time. Weak ties particularly contributed to this activity (vs. being alone or with close friends or family). Involvement with diverse ties or behaviors was linked to improved mood. | • Not applicable | **** |
| Fuentecilla JL, 2020 / USA [37] | To examine links between older adults’ negative social encounters and pain throughout the day and the role of negative mood and closeness with social partners on the links | 313  (56.0%) | 73.96±6.37 | DEWS | • Negative social encounters were associated with greater pain, with negative mood during the same interval acting as a mediator. Negative encounters with less close partners were associated with pain, but not with close partners. | • Not applicable | ***** |
| Huo M, 2020 / USA [38] | To examine whether older adults’ empathy was associated with having encounters with social partners incurring problems and their own well-being | 313  (56.0%) | 73.94±6.38 | DEWS | • Highly empathic older adults noted more social partners with major life problems but did not have increased encounters. Encounters with these partners worsened the mood of older adults, but this effect weakened or reversed among those with higher empathy. | • Not applicable | ***** |
| Bartlett MY, 2019 / USA [63] | To examine the effect of a daily gratitude exercise on gratitude (i.e., treatment group), loneliness, and health (i.e., subjective well-being, and health symptoms) | 36  (80.0%) | 73 (SE=6.43) | Specific name not mentioned | • Gratitude predicted loneliness and health, with the cumulative effect of loneliness and health symptoms being stronger in the treatment group. Loneliness mediated the associations between gratitude and subjective well-being and between gratitude and health symptoms only for the treatment group. | (Loneliness)  • Gratitude | *** |
| Birditt KS, 2019 / USA [39] | To examine solitude as the lack of social contact, its implications for daily emotional well-being, and whether these links vary by the quality of social networks | 313  (55.6%) | 73.94±6.38 | DEWS | • 11% of the participants reported having no social contact (i.e., solitude). Solitude predicted lower negative and positive affect, with the solitude-negative affect being stronger in a conflictual network. | • Not applicable | ***** |
| Huo M, 2019 / USA [40] | To examine how older adults' empathy influences their daily support exchanges with social partners and their daily mood | 293  (55.0%) | 74.05±6.57 | DEWS | • More empathic older adults provided each type of support more often and received more emotional support than less empathic older adults. Empathy moderated the association between support provision and daily mood, and specifically, the positive mood of less empathic older adults was influenced by the type of support they provided. | (Provision and receipt of support)  • Empathy^a^ | ***** |
| Jiang D, 2019 / Hong Kong and Canada [67] | To investigate affective experiences in solitude in daily life and their relationship with overall well-being, while examining the moderating effects of culture and immigration | 162  (60.0%) | 71.67±5.30 | Combined datasets from Canada and Hong Kong | • Positive affect in solitude was more positively associated with well-being in older adults who had immigrated to Canada (aged 75 years or above, immigrated Caucasians and immigrated East Asians) as compared to those who had aged in place. | • Not applicable | **** |
| Zhaoyang R, 2018 / USA [58] | To explore how the intraindividual variability of different aspects of social interactions varies by age | 173^e^  (51.0%) | 49.39±16.99 | Specific name not mentioned^f^ | • The intraindividual variability of interaction frequency with peripheral partners, partner diversity, and interaction quality (positivity and negativity) was lower among older adults than younger adults. | (Social interaction; frequency, partner type, diversity, social interaction quality)  • Age^a^ | ***** |
| Chui H, 2014 / Australia [62] | To examine the associations between the presence of different types of social partners and momentary positive/negative affect of oldest-old adults | 74  (68.0%) | 88.65±3.04 | ADuLTS^j^ Study and other sources | • Spending time alone was associated with lower positive affect, while being with family and friends had a more positive affect. Women and individuals with chronic health conditions reported more negative affect when with spouses, whereas lonelier individuals reported more positive affect when with spouses. | • Not applicable | ***** |
| Heo J, 2010 / USA [66] | To investigate how serious leisure, individual differences, social context, and location contribute to the flow of experience (i.e., challenges and skills regarding activity) in the daily lives of older adults | 19  (68.4%) | 72.0±6.0 | Not applicable | • Retirement was negatively associated with flow experience, and being at home was positively associated with flow experience. Other variables (i.e., social context, serious leisure), as well as age and gender, were not significantly associated with the flow experience. | • Not applicable | *** |
| Rook KS, 2001 / USA [65] | To examine the associations between positive/negative daily social exchanges and well-being (daily mood, loneliness, depression) | 129  (64.0%) | 70.27 | Specific name not mentioned^g^ | • Negative social exchanges were associated with lower positive and greater negative moods, while positive exchanges were only associated with greater positive moods.  • Increased daily negative interactions were associated with higher depression, and positive interactions offset the adverse impact of negative exchanges and lowered the likelihood of depression. | • Not applicable | *** |

Abbreviations: SD, Standard Deviation; MMAT, Mixed Methods Assessment Tool; CHART, Chicago Health and Activity Space in Real-Time; EAS, Einstein Aging Study; DEWS, Daily Experiences and Well-being Study; CCSHC, Continued Care Senior Housing Community; MUAWO, Media Use and Well-Being of Older Adults; MCI, Mild Cognitive Impairment; SE, Standard Error; ADuLTS, Australia Longitudinal Study of Ageing (ALSA) Daily-Life Time-Sampling; CRP, C-reactive protein.

^a^ Results measured at baseline

^b^ Study on digitalization and social lives of older adults.

^c^ Study on prosociality in the daily lives.

^d^ Sample consisted of 477 adults aged 21 and older, of which 147 are older adults.

^e^ Sample consisted of all adults aged 20-80 years, of which 173 are older adults.

^f^ Larger longitudinal measurement burst study of cognition, health, and aging across the lifespan.

^g^ Larger longitudinal study to examine the effects on older adults' mental and physical health of assuming a significant volunteer role.
