# Supplementary material for: Ecological Momentary Assessment to Measure Social Connectedness in Older Adults: Integrative Review
Source: J Med Internet Res. 2025 Jun 17;27:e66324. doi: 10.2196/66324 (PMC12214698; doi:10.2196/66324)
Supplement: Multimedia Appendix 4 [file jmir_v27i1e66324_app4.docx]

Multimedia Appendix 4. Inclusion and exclusion criteria by data source

| First author, year | Inclusion criteria | Exclusion criteria | Eligibility by parent or marker study |
| --- | --- | --- | --- |
|  |  |  |  |
| Compernolle EL, 2021 [48] | • 65 years or older  • Community-dwelling | • Not reported | • Data source: CHART  • Inclusion criteria were healthy older adults aged 65 and older living in 10 diverse city neighborhoods in Chicago [68]. |
| Compernolle EL, 2022 [49] | • 65 years or older  • Community-dwelling | • Not reported |  |
| Goldman AW, 2023 [50] | • Community-dwelling | • Not reported |  |
| Goldman AW, 2023 [51] | • 65 years or older  • Community-dwelling | • Not reported |  |
| Ferguson G, 2024 [41] | • 70 years or older  • Community-dwelling | • Not reported | • Data source: EAS  • Inclusion criteria included being at least 70 years of age; Bronx residents; non-institutionalized; and English-speaking. Exclusion criteria included visual or auditory impairments that preclude neuropsychological testing; active psychiatric symptomatology that interferes with the ability to complete assessments; and non-ambulatory status [69]. |
| Jang H, 2024 [42] | • 70 years or older  • Community-dwelling  • Ambulatory  • Fluent in English | • Hearing or vision loss  • Substance abuse  • Severe psychiatric symptoms  • Chronic medical use of opioids or glucocorticoids  • Recent cancer treatment  • Diagnosed with dementia |  |
| Kang JE, 2024 [43] | • 70 years or older  • Ambulatory  • Fluent in English  • Community-dwelling | • Visual or auditory impairments  • Experiencing psychiatric symptoms that hindered assessments  • Diagnosed with dementia |  |
| Van Bogart, 2023 [44] | • 70 years or older  • Community-dwelling  • Ambulatory  • Fluent in English | • Diagnosed with dementia |  |
| Van Bogart K, 2021 [45] | • 70 years or older  • Ambulatory  • Fluent in English  • Community-dwelling | • Did not complete data on loneliness or inflammation  • Taking strong anti-inflammatory or immunosuppressant medications |  |
| Zhaoyang R, 2022 [46] | • 70 years or older  • Community-dwelling  • Ambulatory  • English-speaking | • Not reported |  |
| Zhaoyang R, 2021 [15] | • 70 years or older  • Community-dwelling  • Ambulatory  • English-speaking | • Hearing or vision loss  • Current substance abuse  • Severe psychiatric symptoms that may interfere with testing  • Chronic medicinal use (i.e., opioids or glucocorticoids, treatment for cancer within the last 12 months)  • Diagnosed with dementia |  |
| Zhaoyang R, 2021 [47] | • 70 years or older  • Community-dwelling  • Ambulatory  • English-speaking | • Hearing or vision loss  • Current substance abuse  • Severe psychiatric symptoms that may interfere with testing  • Alcohol or substance abuse  • Chronic medicinal use (i.e., opioids or glucocorticoids, treatment for cancer within the last 12 months)  • Institutionalized, or non-ambulatory  • Being non-English speaking  • Diagnosed with dementia using DSM-IV criteria |  |
| Fingerman KL, 2024 [26] | • 65 years or older  • Community-dwelling | • Not reported | • Data source: DEWS  • Inclusion criteria included being aged 65 years and older; living in the greater Austin, Texas, Metropolitan Statistical Area; residing at home; and not working full-time [70]. |
| Zhang S, 2024 [27] | • 65 years or older  • Community-dwelling | • Not reported |  |
| Zhou ZX, 2023 [28] | • 65 years or older  • Community-dwelling | • Not reported |  |
| Kim YK, 2022 [29] | • 65 years or older  • Community-dwelling | • Not reported |  |
| Ng YT, 2022 [30] | • 65 years or older  • Community-dwelling  • Not working full-time for pay | • Not reported |  |
| Zhang S, 2022 [31] | • 65 years or older | • Not reported |  |
| Fingerman KL, 2021 [32] | • 65 years or older  • Community-dwelling  • Retirement (i.e., working for pay 20 or fewer hours a week) | • Individuals who had disabilities in activities of daily living |  |
| Huo M, 2021 [33] | • 65 years or older  • Community-dwelling,  • Retirement (i.e., work fewer than 20 hours per week) | • Not reported |  |
| Ng YT, 2021 [34] | • 65 years or older  • Community-dwelling  • Not working full-time for pay | • Not reported |  |
| Birditt KS, 2020 [35] | • 65 years or older  • Community-dwelling  • Not working more than 20 hours a week | • Not reported |  |
| Fingerman KL, 2020 [36] | • 65 years or older  • Community-dwelling | • Not reported |  |
| Fuentecilla JL, 2020 [37] | • 65 years or older  • Community-dwelling | • Not reported |  |
| Huo M, 2020 [38] | • 65 years or older  • Community-dwelling  • Not have cognitive impairment  • Not employed for pay over 20 hours a week | • Not reported |  |
| Birditt KS, 2019 [39] | • 65 years or older  • Community-dwelling  • Retirement (i.e., working for pay 20 or fewer hours a week) | • Not reported |  |
| Huo M, 2019 [40] | • 65 years or older  • Community-dwelling  • Not employed full-time | • Not reported |  |
| Badal VD, 2022 [60] | • 65 years or older  • Community-dwelling  • Smartphone access to receive daily text messages and surveys | • Dementia, major mental illness or other conditions  • Unable to read and write English | • Data source: CCSHC study's pool  • Inclusion criteria included English-speaking individuals; aged 65 years or older; being able to complete study assessments; and without known diagnosis of dementia or any other disabling illness [71]. |
| Hülür G, 2024 [52] | • 65 years or older  • Being fluent in German  • Using digital devices to communicate  • Having sufficient hearing and vision | • Not reported | • Data source: Specific name not mentioned (study on digitalization and social lives of older adults)  • Inclusion criteria included being 65 years or older; using digital devices to communicate; having sufficient vision and hearing; and being fluent in German [72]. |
| Luo MX, 2024 [53] | • 65 years or older  • Being fluent in German  • Using digital devices to communicate  • Having sufficient hearing and vision | • Not reported |  |
| Luo M, 2022 [54] | • 65 years or older  • Being fluent in German  • Using digital devices to communicate  • Having sufficient hearing and vision | • Not reported |  |
| Luo M, 2022 [55] | • 65 years or older  • Being fluent in German  • Using digital devices to communicate  • Having sufficient hearing and vision | • Not reported |  |
| Macdonald B, 2021 [56] | • 65 years or older  • Being fluent in German  • Ability to use digital devices to communicate  • Having sufficient hearing and vision | • Not reported |  |
| Wallimann M, 2024 [59] | • 65 years or older  • Being fluent in German  • Having the ability to handle a book-sized mobile device and read newspaper-sized print  • No diagnosed neurodegenerative disease or physical impairments affecting hand or finger mobility | • Not reported | • Not applicable |
| Mann AS, 2022 [61] | • 60 years or older  • Holding leadership positions in prosocial programs or investing high numbers of hours in formal volunteering (at least 5 hours per week) | • Not reported | • Data source: Specific name not mentioned (study on prosociality in daily lives) [61] |
| Pfund GN, 2022 [64] | • Not reported | • Not reported | • Data source: MUAWO  • Data were collected from a convenience sample of older adults aged 60 years living in Switzerland [73] |
| Junghaenel DU, 2021 [57] | • 21 years or older  • Residence in the Central or Eastern time zone  • Being fluent in English  • Access to a computer with high-speed Internet at home  • No difficulty reading a computer screen  • No upcoming major events that could interfere with daily assessments  • No night shift work  • Willing to participate for 25 consecutive days | • Not reported | • Not applicable |
| Bartlett MY, 2019 [63] | • Not reported | • Not reported | • Not applicable |
| Jiang D, 2019 [67] | • Not reported | • Not reported | • Data source: Combining two datasets from Vancouver, Canada and Hong Kong, China  • Canadian data set: Eligibility requirements included being 65 years or older; able to read newspaper sized print and to hear an alarm clock; able to speak and understand English, Cantonese, or Mandarin; free of any health conditions for which exercise is contraindicated; and free of any neurodegenerative disease or brain dysfunction [74].  • Hong Kong data set: The procedure for collecting data in Hong Kong was similar to that used in Canada [not presented]. |
| Zhaoyang R, 2018 [58] | • 20-80 years old  • Being fluent in English  • Physical ability to operate palmtop computer  • Absence of major cognitive impairment | • Not reported | • Data source: Specific name not mentioned (study on cognition, health, and aging across the lifespan) [not presented] |
| Chui H, 2014 [62] | • Vision and hearing sufficient to independently complete diaries  • Mini-Mental State Examination ≥ 24 | • Not reported | • Data source: combining ALSA and other sources  • ALSA: Individuals aged 65 and older and living in the community or in residential care were eligible [75].  • Other sources: Community-dwelling older adults recruited from multiple sources, details not described [not presented]. |
| Heo J, 2010 [66] | • 60 years or older | • Not reported | • Not applicable |
| Rook KS, 2001 [65] | • Not reported | • Not reported | • Data source: Specific name not mentioned (study to examining the effects on older adults' mental and physical health of assuming a significant volunteer role) [not presented] |

Abbreviations: CHART, Chicago Health and Activity Space in Real-Time; EAS: Einstein Aging Study; DEWS, Daily Experiences and Well-being Study; CCSHC, Continued Care Senior Housing Community; MUAWO, Media Use and Well-Being of Older Adults; ALSA, Australia Longitudinal Study of Ageing.

References

15. Zhaoyang R, Scott SB, Martire LM, Sliwinski MJ. Daily social interactions related to daily performance on mobile cognitive tests among older adults. PLoS One 2021;16(8):e0256583. [doi:10.1371/journal.pone.0256583] [PMID:34437609]

26. Fingerman KL, Zhou Z, Huo M, Luong G, Birditt KS. Enduring bonds: duration and contact in close relationships in late Life. Gerontologist 2024;64(4). [doi:10.1093/geront/gnad091] [PMID:37434403]

27. Zhang S, Zhou Z, Fingerman KL, Birditt KS. Loneliness and mode of social contact in late life. J Gerontol B Psychol Sci Soc Sci 2024;79(9). [doi:10.1093/geronb/gbae115] [PMID:39192673]

28. Zhou Z, Zhang S, Kim YK, Birditt KS, Fingerman KL. Need to belong, daily social engagement, and transient loneliness in late life. Journal of Social and Personal Relationships 2023;41(1):115-136. [doi:10.1177/02654075231211617]

29. Kim YK, Fingerman KL. Daily social media use, social ties, and emotional well-being in later life. J Soc Pers Relat 2022;39(6):1794-1813. [doi:10.1177/02654075211067254] [PMID:37727534]

30. Ng YT, Huo M, Han SH, Birditt KS, Fingerman KL. Older adult's marital status, conversation frequency, and well-being in everyday life. J Gerontol B Psychol Sci Soc Sci 2022;77(3):499-512. [doi:10.1093/geronb/gbab112] [PMID:34159387]

31. Zhang S, Kim YK, Fingerman KL, Birditt KS, Charles ST. Narcissism, social experiences, and mood in late life. J Gerontol B Psychol Sci Soc Sci 2022;77(8):1442-1453. [doi:10.1093/geronb/gbac019] [PMID:35092423]

32. Fingerman KL, Ng YT, Huo M, Birditt KS, Charles ST, Zarit S. Functional limitations, social integration, and daily activities in late life. J Gerontol B Psychol Sci Soc Sci 2021;76(10):1937-1947. [doi:10.1093/geronb/gbab014] [PMID:33460446]

33. Huo M, Ng YT, Fuentecilla JL, Leger K, Charles ST. Positive encounters as a buffer: pain and sleep disturbances in older adults' everyday Lives. J Aging Health 2021;33(1-2):75-85. [doi:10.1177/0898264320958320] [PMID:32897128]

34. Ng YT, Huo M, Gleason ME, Neff LA, Charles ST, Fingerman KL. Friendships in old age: daily encounters and emotional well-being. J Gerontol B Psychol Sci Soc Sci 2021;76(3):551-562. [doi:10.1093/geronb/gbaa007] [PMID:31943103]

35. Birditt KS, Polenick CA, Luong G, Charles ST, Fingerman KL. Daily interpersonal tensions and well-being among older adults: the role of emotion regulation strategies. Psychol Aging 2020;35(4):578-590. [doi:10.1037/pag0000416] [PMID:31670541]

36. Fingerman KL, Huo M, Charles ST, Umberson DJ. Variety is the spice of late life: social integration and daily activity. J Gerontol B Psychol Sci Soc Sci 2020;75(2):377-388. [doi:10.1093/geronb/gbz007] [PMID:30783671]

37. Fuentecilla JL, Huo M, Birditt KS, Charles ST, Fingerman KL. Interpersonal tensions and pain among older adults: the mediating role of negative mood. Res Aging 2020;42(3-4):105-114. [doi:10.1177/0164027519884765] [PMID:31709931]

38. Huo M, Fuentecilla JL, Birditt KS, Fingerman KL. Does empathy have a cost? older adults and social partners experiencing problems. Gerontologist 2020;60(4):617-627. [doi:10.1093/geront/gnz065] [PMID:31152585]

39. Birditt KS, Manalel JA, Sommers H, Luong G, Fingerman KL. Better off alone: daily solitude is associated with lower negative affect in more conflictual social networks. Gerontologist 2019;59(6):1152-1161. [doi:10.1093/geront/gny060] [PMID:29924314]

40. Huo M, Fuentecilla JL, Birditt KS, Fingerman KL. Older adults' empathy and daily support exchanges. J Soc Pers Relat 2019;36(11-12):3814-3834. [doi:10.1177/0265407519837372] [PMID:31814654]

41. Ferguson G, Ali S, Pasquini G, et al. Daily and trait personality assessments’ relationship with daily experiences of stress risk and resilience factors. Journal of Research in Personality 2024;109:104452. [doi:https://doi.org/10.1016/j.jrp.2023.104452]

42. Jang H, Hill NL, Turner JR, Bratlee-Whitaker E, Jeong M, Mogle J. Poor-quality daily social encounters, daily stress, and subjective cognitive decline among older adults. Innovation in Aging 2024;8(6). [doi:10.1093/geroni/igae038]

43. Kang JE, Graham-Engeland JE, Martire LM, Almeida DM, Sliwinski MJ. Short-term coupling associations between state loneliness and cognitive performance in daily life among older adults. J Gerontol B Psychol Sci Soc Sci 2024;79(10). [doi:10.1093/geronb/gbae134] [PMID:39105303]

44. Van Bogart K, Scott SB, Harrington KD, Felt JM, Sliwinski MJ, Graham-Engeland JE. Examining the bidirectional nature of loneliness and anxiety among older adults in daily life. J Gerontol B Psychol Sci Soc Sci 2023;78(10):1676-1685. [doi:10.1093/geronb/gbad105] [PMID:37527478]

45. Van Bogart K, Engeland CG, Sliwinski MJ, et al. The association between loneliness and inflammation: findings from an older adult sample. Front Behav Neurosci 2021;15:801746. [doi:10.3389/fnbeh.2021.801746] [PMID:35087386]

46. Zhaoyang R, Harrington KD, Scott SB, Graham-Engeland JE, Sliwinski MJ. Daily social interactions and momentary loneliness: the role of trait loneliness and neuroticism. J Gerontol B Psychol Sci Soc Sci 2022;77(10):1791-1802. [doi:10.1093/geronb/gbac083] [PMID:35758315]

47. Zhaoyang R, Sliwinski MJ, Martire LM, Katz MJ, Scott SB. Features of daily social interactions that discriminate between older adults with and without mild cognitive impairment. J Gerontol B Psychol Sci Soc Sci 2021;79(4). [doi:10.1093/geronb/gbab019] [PMID:33528558]

48. Compernolle E, Finch LE, Hawkley LC, Cagney KA. Momentary loneliness among older adults: contextual differences and their moderation by gender and race/ethnicity. Soc Sci Med 2024;350:116743. [doi:10.1016/j.socscimed.2024.116743] [PMID:38522965]

49. Compernolle EL, Finch LE, Hawkley LC, Cagney KA. Home alone together: Differential links between momentary contexts and real-time loneliness among older adults from Chicago during versus before the COVID-19 pandemic. Soc Sci Med 2024;346:116744. [doi:10.1016/j.socscimed.2024.116744] [PMID:38494392]

50. Goldman AW, Compernolle EL. Personal network size and social accompaniment: protective or risk factor for momentary loneliness, and for whom? Soc Ment Health 2023;13(1):23-44. [doi:10.1177/21568693221142336] [PMID:38665906]

51. Goldman A, York Cornwell E. Stand by me: social ties and health in real-time. Socius 2023;9. [doi:10.1177/23780231231171112] [PMID:37822581]

52. Hülür G, Luo M, Macdonald B, Grünjes CE. The perceived quality of social interactions differs by modality and purpose: an event-contingent experience sampling study with older adults. Journal of Social and Personal Relationships 2024;41(4):794-821. [doi:10.1177/02654075231215269]

53. Luo M, Yordanova K, Macdonald B, Hülür G. Routineness of social interactions is associated with higher affective well-being in older adults. J Gerontol B Psychol Sci Soc Sci 2024;79(6). [doi:10.1093/geronb/gbae057] [PMID:38595036]

54. Luo M, Macdonald B, Hülür G. Not "the more the merrier": diminishing returns to daily cace-to-face social interaction frequency for well-being in older age. J Gerontol B Psychol Sci Soc Sci 2022;77(8):1431-1441. [doi:10.1093/geronb/gbac010] [PMID:35077534]

55. Luo M, Pauly T, Röcke C, Hülür G. Alternating time spent on social interactions and solitude in healthy older adults. Br J Psychol 2022;113(4):987-1008. [doi:10.1111/bjop.12586] [PMID:35957493]

56. Macdonald B, Luo M, Hülür G. Daily social interactions and well-being in older adults: The role of interaction modality. Journal of Social and Personal Relationships 2021;38(12):3566-3589. [doi:10.1177/02654075211052536]

57. Junghaenel DU, Broderick JE, Schneider S, et al. Explaining age differences in the memory-experience gap. Psychol Aging 2021;36(6):679-693. [doi:10.1037/pag0000628] [PMID:34516172]

58. Zhaoyang R, Sliwinski MJ, Martire LM, Smyth JM. Age differences in adults' daily social interactions: an ecological momentary assessment study. Psychol Aging 2018;33(4):607-618. [doi:10.1037/pag0000242] [PMID:29708385]

59. Wallimann M, Peleg S, Pauly T. Time-savoring moderates associations of solitude with depressive mood, loneliness, and somatic symptoms in older adults' daily life. Appl Psychol Health Well Being 2024;16(3):1497-1515. [doi:10.1111/aphw.12538] [PMID:38520051]

60. Badal VD, Lee EE, Daly R, et al. Dynamics of loneliness among older adults during the COVID-19 pandemic: pilot study of ecological momentary assessment with network analysis. Front Digit Health 2022;4:814179. [doi:10.3389/fdgth.2022.814179] [PMID:35199099]

61. Mann AS, Boeder J, Tse DCK, Graham L, Nakamura J. Solitary prosociality in later life: an experience sampling study. Res Aging 2022;44(9-10):724-733. [doi:10.1177/01640275211062124] [PMID:35271402]

62. Chui H, Hoppmann CA, Gerstorf D, Walker R, Luszcz MA. Social partners and momentary affect in the oldest-old: the presence of others benefits affect depending on who we are and who we are with. Dev Psychol 2014;50(3):728-740. [doi:10.1037/a0033896] [PMID:23895170]

63. Bartlett MY, Arpin SN. Gratitude and loneliness: Enhancing health and well-being in older adults. Res Aging 2019;41(8):772-793. [doi:10.1177/0164027519845354] [PMID:31043126]

64. Pfund GN, Hofer M, Allemand M, Hill PL. Being social may be purposeful in older adulthood: a measurement burst design. Am J Geriatr Psychiatry 2022;30(7):777-786. [doi:10.1016/j.jagp.2021.11.009] [PMID:34924274]

65. Rook KS. Emotional health and positive versus negative social exchanges: a daily diary analysis. Applied Developmental Science 2001;5(2):86-97. [doi:10.1207/S1532480XADS0502_4]

66. Heo J, Lee Y, Pedersen PM, McCormick BP. Flow experience in the daily lives of older adults: an analysis of the interaction between flow, individual differences, serious leisure, location, and social context. Can J Aging 2010;29(3):411-423. [doi:10.1017/s0714980810000395] [PMID:20707938]

67. Jiang D, Fung HH, Lay JC, Ashe MC, Graf P, Hoppmann CA. Everyday solitude, affective experiences, and well-being in old age: the role of culture versus immigration. Aging Ment Health 2019;23(9):1095-1104. [doi:10.1080/13607863.2018.1479836] [PMID:30621431]

68. English N, Zhao C, Brown KL, Catlett C, Cagney K. Making sense of sensor data: how local environmental conditions add value to social science research. Soc Sci Comput Rev 2022;40(1):179-194. [doi:10.1177/0894439320920601] [PMID:35400811]

69. Katz MJ, Lipton RB, Hall CB, Zimmerman ME, Sanders AE, Verghese J, Dickson DW, Derby CA. Age-specific and sex-specific prevalence and incidence of mild cognitive impairment, dementia, and alzheimer dementia in blacks and whites: a report from the einstein aging study. Alzheimer Dis Assoc Disord 2012; 26(4):335-343. [doi:10.1097/WAD.0b013e31823dbcfc] [PMID:22156756]

70. Fingerman K, Charles S, Birditt K. Inter-University Consortium for Political and Social Research distributor. Daily experiences and well-being in late life, Austin, Texas, 2016-2017. URL: https://www.icpsr.umich.edu/web/NACDA/studies/38570 [accessed 2022-11-28]

71. Jeste DV, Glorioso D, Lee EE, Daly R, Graham S, Liu J, Paredes AM, Nebeker C, Tu XM, Twamley EW, Van Patten R, Yamada Y, Depp C, Kim H. Study of independent living residents of a continuing care senior housing community: sociodemographic and clinical associations of cognitive, physical, and mental health. Am J Geriatr Psychiatry 2019; 27(9):895-907. [doi:10.1016/j.jagp.2019.04.002] [PMID:31078382]

72. Macdonald B, Hülür G. Digitalization and the social lives of older adults: protocol for a microlongitudinal study. JMIR Res Protoc 2020;9(10):e20306. [doi:10.2196/20306] [PMID:33001037]

73. Hofer M, Birrer A, Eden A, Seifert A. Daily TV use and meaning in life among older adults: the moderating role of selective and compensatory TV use. Mass Communication and Society 2022; 27(5):1006-1027. [doi:10.1080/15205436.2022.2135447]

74. Hoppmann CA, Lee JCM, Ziegelmann JP, Graf P, Khan KM, Ashe MC. Precipitation and physical activity in older adults: the moderating role of functional mobility and physical activity intentions. J Gerontol B Psychol Sci Soc Sci 2017;72(5):792-800. [doi:10.1093/geronb/gbv107] [PMID:26707498]

75. Luszcz MA, Giles LC, Anstey KJ, Browne-Yung KC, Walker RA, Windsor TD. Cohort profile: the Australian longitudinal study of ageing (ALSA). Int J Epidemiol 2016; 45(4):1054-1063. [doi:10.1093/ije/dyu196] [PMID:25468824]
