# Supplementary material for: Ecological Momentary Assessment to Measure Social Connectedness in Older Adults: Integrative Review
Source: J Med Internet Res. 2025 Jun 17;27:e66324. doi: 10.2196/66324 (PMC12214698; doi:10.2196/66324)
Supplement: Multimedia Appendix 5 [file jmir_v27i1e66324_app5.docx]

Multimedia Appendix 5. Quality evaluation of the selected studies

| First author, year | Quality criteria | | | | | |
| --- | --- | --- | --- | --- | --- | --- |
|  | 1. Are the participants representative of the target population? | 1. Are measurements appropriate regarding both the outcome and intervention (or exposure)? | 1. Are there complete outcome data? | 1. Are the confounders accounted for in the design and analysis? | 1. During the study period, is the intervention administered (or exposure occurred) as intended? | Overall quality scores |
|  |  |  |  |  |  |  |
| Compernolle EL, 2024 [48] | Yes | Yes | Yes | Yes | Yes | ***** |
| Compernolle EL, 2024 [49] | Yes | Yes | Yes | Yes | Yes | ***** |
| Ferguson G, 2024 [41] | Yes | Yes | Yes | Yes | Yes | ***** |
| Fingerman KL, 2024 [26] | Yes | Yes | Yes | Yes | Yes | ***** |
| Hülür G, 2024 [52] | Yes | Yes | Yes | Yes | Yes | ***** |
| Jang H, 2024 [42] | Yes | Yes | Yes | Yes | Yes | ***** |
| Kang JE, 2024 [43] | Yes | Yes | Yes | Yes | Yes | ***** |
| Luo MX, 2024 [53] | Yes | Yes | Yes | Yes | Yes | ***** |
| Wallimann M, 2024 [59] | Yes | Yes | Yes | Yes | Yes | ***** |
| Zhang S, 2024 [27] | Yes | Yes | Yes | Yes | Yes | ***** |
| Zhou ZX, 2023 [28] | Yes | Yes | Yes | Yes | Yes | ***** |
| Goldman AW, 2023 [50] | Yes | Yes | No | Yes | Yes | **** |
| Goldman AW, 2023 [51] | Yes | Yes | No | Yes | Yes | **** |
| Van Bogart, 2023 [44] | Yes | Yes | Yes | Yes | Yes | ***** |
| Badal VD, 2022 [60] | Yes | Yes | Cannot tell | Cannot tell | Yes | *** |
| Kim YK, 2022 [29] | Yes | Yes | Yes | Yes | Yes | ***** |
| Luo M, 2022 [54] | Yes | Yes | Yes | Yes | Yes | ***** |
| Luo M, 2022 [55] | Yes | Yes | Yes | Yes | Yes | ***** |
| Mann AS, 2022 [61] | Yes | Yes | No | Yes | Yes | **** |
| Ng YT, 2022 [30] | Yes | Yes | Yes | Yes | Yes | ***** |
| Pfund GN, 2022 [64] | Cannot tell | Yes | Cannot tell | Yes | Yes | *** |
| Van Bogart K, 2021 [45] | Yes | Yes | No | Yes | Yes | **** |
| Zhang S, 2022 [31] | Yes | Yes | Yes | Yes | Yes | ***** |
| Zhaoyang R, 2022 [46] | Yes | Yes | Yes | Yes | Yes | ***** |
| Fingerman KL, 2021 [32] | Yes | Yes | Yes | Yes | Yes | ***** |
| Huo M, 2021 [33] | Yes | Yes | Yes | Yes | Yes | ***** |
| Junghaenel DU, 2021 [57] | No | Yes | Yes | Yes | Yes | **** |
| Macdonald B, 2021 [56] | Yes | Yes | Yes | Yes | Yes | ***** |
| Ng YT, 2021 [34] | Yes | Yes | Yes | Yes | Yes | ***** |
| Zhaoyang R, 2021 [15] | Yes | Yes | Cannot tell | Yes | Yes | **** |
| Zhaoyang R, 2021 [47] | Yes | Yes | Yes | Yes | Yes | ***** |
| Birditt KS, 2020 [35] | Yes | Yes | Yes | Yes | Yes | ***** |
| Fingerman KL, 2020 [36] | Cannot tell | Yes | Yes | Yes | Yes | **** |
| Fuentecilla JL, 2020 [37] | Yes | Yes | Yes | Yes | Yes | ***** |
| Huo M, 2020 [38] | Yes | Yes | Yes | Yes | Yes | ***** |
| Bartlett MY, 2019 [63] | No | Yes | Yes | Yes | No | *** |
| Birditt KS, 2019 [39] | Yes | Yes | Yes | Yes | Yes | ***** |
| Huo M, 2019 [40] | Yes | Yes | Yes | Yes | Yes | ***** |
| Jiang D, 2019 [67] | Cannot tell | Yes | Yes | Yes | Yes | **** |
| Zhaoyang R, 2018 [58] | Yes | Yes | Yes | Yes | Yes | ***** |
| Chui H, 2014 [62] | Yes | Yes | Yes | Yes | Yes | ***** |
| Heo J, 2010 [66] | Cannot tell | Yes | Yes | No | Yes | *** |
| Rook KS, 2001 [65] | No | Yes | Yes | No | Yes | *** |
