# Supplementary material for: Ecological Momentary Assessment to Measure Social Connectedness in Older Adults: Integrative Review
Source: J Med Internet Res. 2025 Jun 17;27:e66324. doi: 10.2196/66324 (PMC12214698; doi:10.2196/66324)
Supplement: Multimedia Appendix 6 [file jmir_v27i1e66324_app6.docx]

Multimedia Appendix 6. EMA of social connectedness and other contextual variables

| First author, year | Social connectedness | | | Context | Interaction type | Others |
| --- | --- | --- | --- | --- | --- | --- |
|  | Structural | Functional | Quality |  |  |  |
|  |  |  |  |  |  |  |
| Compernolle EL, 2024 [48] | Social accompaniment (with whom) | Loneliness |  | Location | In-person |  |
| Compernolle EL, 2024 [49] | Social accompaniment (with whom) | Loneliness |  | Location | In-person |  |
| Ferguson G, 2024 [41] | Social interaction |  |  |  | In-person, phone/computer, texting | Stress experience, daily personality |
| Fingerman KL, 2024 [26] | Encounters with social partners^a^ (social partner contacts) |  | Interpersonal stressful encounters |  | In-person, telephone, texting or other electronic communication contact (focus on in-person and telephone contact) | Positive and negative mood |
| Hülür G, 2024 [52] | Social interaction (modality and purpose) |  | Interaction quality |  | In-person, telephone, text-based digital interaction | Social relatedness, calmness, eudaimonic |
| Jang H, 2024 [42] | Social encounter |  | Quality of social encounters |  | Not mentioned | Daily stress |
| Kang JE, 2024 [43] | Solitude (with other or not) | Loneliness |  | Location  Ambulatory cognition | Not mentioned |  |
| Luo MX, 2024 [53] | Social interaction (interaction time, modality and partner type) |  |  | Location | Spoken and text-based interactions^b^ |  |
| Wallimann M, 2024 [59] | Solitude (time spent alone) | Loneliness |  |  | Not mentioned | Time-savoring, depressive mood, somatic symptoms |
| Zhang S, 2024 [27] | Encounters with social partners^a^  (social partner contacts, modality) | Loneliness |  |  | In-person, phone, text, email, social media |  |
| Zhou ZX, 2023 [28] | Encounters with social partners^a^ (social partner contacts, modality) | Loneliness |  | Engagement in groups | In-person, phone |  |
| Goldman AW, 2023 [50] | Social accompaniment (with whom) | Loneliness |  | Location | In-person |  |
| Goldman AW, 2023 [51] | Social accompaniment (with whom) |  |  | Location, Activity type | In-person | Pain, fatigue, stress |
| Van Bogart, 2023 [44] |  | Loneliness |  |  | Not mentioned | Anxiety, depressed state, mood valence |
| Badal VD, 2022 [60] | Social interaction (number of interaction partner) | Loneliness |  | Spending time outdoor, exercise time | Spoken and text-based interactions^b^ | Positive and negative mood |
| Kim YK, 2022 [29] | Social interaction (modality) | Loneliness |  | Social media use | Not mentioned | Positive and negative mood |
| Luo M, 2022 [54] | Social interaction (number of interaction partner) | Loneliness |  |  | In-person, telephone, social media | Positive and negative mood |
| Luo M, 2022 [55] | Social interaction (interaction duration) |  |  |  | Spoken and text-based interactions^b^ |  |
| Mann AS, 2022 [61] | Social interaction (with whom, frequency) |  |  | Activity type | In-person, telecommunication | Positive and negative affect, sense of relatedness |
| Ng YT, 2022 [30] | Encounters with social partners^a^ (social partner contacts) |  |  |  | In-person, phone, text | Positive and negative mood |
| Pfund GN, 2022 [64] | Social interaction |  | Social interaction quality |  | Not mentioned | Daily sense of purpose |
| Van Bogart K, 2021 [45] |  | Loneliness |  |  | Not mentioned |  |
| Zhang S, 2022 [31] | Social encounters (with whom) |  | Social encounters pleasantness,  stressful discussion |  | Not mentioned | Positive and negative mood |
| Zhaoyang R, 2022 [46] | Social interaction (partner type and current status of solitude) | Loneliness | Social interaction quality | Activity type | In-person, online | Negative affect |
| Fingerman KL, 2021 [32] | Encounters with social partners^a^ (social partner contacts) |  |  | Location, Activities type | Not mentioned |  |
| Huo M, 2021 [33] | Encounters with social partners^a^ (social partner contacts) |  | Positive encounters |  | In-person, phone, or text | Pain, sleep disturbance |
| Junghaenel DU, 2021 [57] |  | Loneliness |  |  | Not mentioned | Positive and negative affect, fatigue and pain, anxiety and anger |
| Macdonald B, 2021 [56] | Social interaction (frequency) | Loneliness | Pleasantness of interactions |  | Spoken and text-based interactions^b^ | Positive and negative affect |
| Ng YT, 2021 [34] | Encounters with social partners^a^ (social partner contacts) |  | Pleasantness of the encounter, stressful discussion | Activity type | In-person, phone, text | Positive and negative mood |
| Zhaoyang R, 2021 [15] | Social interaction (frequency and partner type) |  | Social interaction quality | Cognitive function | In-person, phone/computer, text |  |
| Zhaoyang R, 2021 [47] | Social interaction (partner type) |  | Social interaction quality | Activity type | In-person, phone/computer, text |  |
| Birditt KS, 2020 [35] | Social interaction |  | Interpersonal tensions |  | Not mentioned | Positive and negative emotion |
| Fingerman KL, 2020 [36] | Encounters with social partners^a^ (social partner contacts) |  |  | Diverse behaviors, physical activity^c^, sedentary time^c^ | Not mentioned | Positive and negative emotion |
| Fuentecilla JL, 2020 [37] | Encounters with social partners^a^ (social partner contacts) |  | Negative social encounters |  | Not mentioned | Pain, negative mood |
| Huo M, 2020 [38] | Encounters with social partners^a^ (social partner contacts) |  | Stressful discussion; Pleasantness of the encounter |  | Not mentioned | Positive and negative emotions |
| Bartlett MY, 2019 [63] |  | Loneliness |  | Activities of daily living difficulty | Not mentioned | Gratitude, subjective well-being, subjective health, symptom, positive affect |
| Birditt KS, 2019 [39] | Daily solitude^a^ (social partner contacts) |  |  |  | In-person, phone, electronic communication | Positive and negative emotions |
| Huo M, 2019 [40] |  | Support exchange  (provision and receipt of emotional support, instrumental support, and advice) |  |  | Not mentioned | Positive and negative emotions |
| Jiang D, 2019 [67] | Solitude |  |  |  | Not mentioned | Positive and negative affect |
| Zhaoyang R, 2018 [58] | Social interaction (with whom and frequency) |  | Social interaction quality |  | In-person, phone, online |  |
| Chui H, 2014 [62] | Social interaction (with whom) |  |  |  | In-person | Positive and negative affect |
| Heo J, 2010 [66] | Social interaction (with whom) |  |  | Location | Not mentioned | Personal identity in relation to serious leisure, activity-related challenges and skills, |
| Rook KS, 2001 [65] | Social exchange |  | Positive and negative social interactions |  | In-person, telephone |  |

^a^ In the initial interview, participants provided the names of their close social ties using a social convoy measure, which indicates whether they had contact with their 10 closest social partners and up to 6 other social partners.

^b^ Spoken interactions included face-to-face, telephone, and video chat; text-based conversations included text message, e-mail, and letter.

^c^ Indicators using accelerometer assessment.
