# Supplementary material for: Ecological Momentary Assessment to Measure Social Connectedness in Older Adults: Integrative Review
Source: J Med Internet Res. 2025 Jun 17;27:e66324. doi: 10.2196/66324 (PMC12214698; doi:10.2196/66324)
Supplement: Multimedia Appendix 7 [file jmir_v27i1e66324_app7.docx]

Multimedia Appendix 7. Trait-level assessment of social connectedness

| First author, year | **Structural** | **Functional** | **Quality** |
| --- | --- | --- | --- |
|  |  |  |  |
| Compernolle EL, 2024 [48] | • Marital status  • Household composition |  |  |
| Compernolle EL, 2024 [49] | • Marital status  • Living arrangement |  |  |
| Ferguson G, 2024 [41] |  |  |  |
| Fingerman KL, 2024 [26] | • Marital status  • General social integration (social network) |  |  |
| Hülür G, 2024 [52] |  |  |  |
| Jang H, 2024 [42] | • Living arrangement |  |  |
| Kang JE, 2024 [43] | • Marital status  • Living arrangement |  |  |
| Luo MX, 2024 [53] | • Living arrangement |  |  |
| Wallimann M, 2024 [59] | • Living arrangement |  |  |
| Zhang S, 2024 [27] | • Marital status  • Social network size |  |  |
| Zhou ZX, 2023 [28] | • Marital status |  | • Negative social interaction |
| Goldman AW, 2023 [50] | • Social interaction (frequency)  • Social network (size)  • Marital status |  |  |
| Goldman AW, 2023 [51] | • Social interaction (frequency)  • Social network (size)  • Living arrangement |  |  |
| Van Bogart, 2023 [44] | • Marital status  • Living arrangement |  |  |
| Badal VD, 2022 [60] | • Marital status | • Emotional & Instrumental support  • Loneliness | • Negative social interaction |
| Kim YK, 2022 [29] | • Marital status  • Social network structure |  |  |
| Luo M, 2022 [54] | • Social interaction average frequency  • Marital status  • Living arrangement |  |  |
| Luo M, 2022 [55] | • Marital status |  |  |
| Mann AS, 2022 [61] | • Marital status  • Roles in primary prosocial-program |  |  |
| Ng YT, 2022 [30] | • Marital status |  |  |
| Pfund GN, 2022 [64] | • Marital status |  |  |
| Van Bogart K, 2021 [45] | • Marital status  • Living arrangement | • Loneliness |  |
| Zhang S, 2022 [31] | • Marital status |  |  |
| Zhaoyang R, 2022 [46] | • Marital status  • Living arrangement | • Loneliness |  |
| Fingerman KL, 2021 [32] | • Marital status  • General social integration |  |  |
| Huo M, 2021 [33] | • Marital status |  |  |
| Junghaenel DU, 2021 [57] | • Marital status  • Living situation |  |  |
| Macdonald B, 2021 [56] | • Marital status  • Living arrangement |  |  |
| Ng YT, 2021 [34] | • Marital status |  |  |
| Zhaoyang R, 2021 [15] |  |  |  |
| Zhaoyang R, 2021 [47] | • Number of close social relationships  • Contact frequency with social partners  • Marital status  • Living arrangement | • Social support | • Social strain |
| Birditt KS, 2020 [35] | • Marital status  • Living arrangement |  |  |
| Fingerman KL, 2020 [36] | • Marital status |  |  |
| Fuentecilla JL, 2020 [37] | • Marital status |  |  |
| Huo M, 2020 [38] | • Marital status |  |  |
| Bartlett MY, 2019 [63] | • Marital status |  |  |
| Birditt KS, 2019 [39] | • Social network size  • Marital status  • Living arrangement |  | • Social network quality |
| Huo M, 2019 [40] | • Marital status  • Social network (close partners) |  |  |
| Jiang D, 2019 [67] | • Marital status  • Living alone |  |  |
| Zhaoyang R, 2018 [58] | • Marital status  • Living arrangement |  |  |
| Chui H, 2014 [62] | • Marital status | • Loneliness |  |
| Heo J, 2010 [66] | • Marital status  • Living arrangement |  |  |
| Rook KS, 2001 [65] | • Marital status | • Loneliness |  |
