# Supplementary material for: Ecological Momentary Assessment to Measure Social Connectedness in Older Adults: Integrative Review
Source: J Med Internet Res. 2025 Jun 17;27:e66324. doi: 10.2196/66324 (PMC12214698; doi:10.2196/66324)
Supplement: Multimedia Appendix 8 [file jmir_v27i1e66324_app8.docx]

EMA protocols

| Studies;  data source | Prompt design | Definition of moment | Times/day | Duration (days) | Type of device | Training | Approaches to achieve response | Criteria for valid EMA response |
| --- | --- | --- | --- | --- | --- | --- | --- | --- |
|  |  |  |  |  |  |  |  |  |
| Compernolle et al [48,49], Goldman and Compernolle [50], Goldman and Cornwell [51]; CHART [68] | Quasi-random basis within each 2-hour interval for 5-time blocks from 8am until 8pm (2 hours/each) | At the time of each ping | 5 | 7days/each wave (3 waves^a^, 5 to 6 months apart) | Study-provided smartphone (with GPS tracking) | Tutorial on how to use the smartphone | Reminder alerts 10 and 20 minutes via app after the initial ping | EMA responses exceeding 30 minutes to complete or initiated beyond 30 minutes after receiving the ping were excluded. |
| Zhaoyang et al [15], Ferguson et al [41], Jang et al [42], Kang et al [43], Van Bogart et al [44,45], Zhaoyang et al [46,47];  EAS [69] | Mixed: (1) once at the end-of-day, (2) quasi-random basis with approximately 3.5 hours apart based on participants’ self-reported wake schedules | Mixed: (1) since last EMA (social interaction), (2) at the beep time^b^ | 5 | 14 | Study-provided smartphone | Training on study protocol, use of the smartphone, and practice for 2 days | Not described | Not described |
| Fingerman et al [26,32,36], Zhang et al [27,31], Zhou et al [28], Kim and Fingerman [29], Ng et al [30,34], Huo et al [33,38,40], Birditt et al [35,39], Fuentecilla et al [37];  DEWS by Fingerman et al [70] | Quasi-random basis with 3 hours apart based on each participant’s self-reported wake time and bedtime | Mixed: (1) since the last EMA (social interaction), (2) end-of-day survey^c^ | 6 | 5–6 (3–4 weekdays and 2 weekend days) | Study-provided smartphone (with EAR), wristwatch | Training for older adults unfamiliar with the device | Not described | Not described |
| Badal et al [60];  CCSHC study by Jeste et al [71] | Quasi-random basis with minimum 4 hours apart | During the previous 2 hours | 3 | 7 | Personal smartphone | Not described | When participants missed three consecutive surveys, research staff contacted them to address any technical difficulties or adherence issues. | Responses completed 1 hour before the next scheduled survey |
| Hülür et al [52], Luo et al [53,54,55], Macdonald et al [56];  not specific name (Macdonald et al [72]) | Mixed: (1) for each conversation, (2) once at the end of the day | Each social interaction | 1. all events/day, (2) once at the end of the day | 21 | Study-provided smartphone | Not described | Not described | Not described |
| Wallimann et al [59];  not applicable | Once at a self-determined bedtime from 6pm to 3am | Since getting up | 1 | 14 | Personal smartphone/tablet or provided device | Not described | Automatic reminders at 8.30 pm and 9 pm | Not described |
| Mann et al [61];  not specific name | Random basis | At each signal | 6 | 7 | Not described | Not described | Not described | Responses completed within 30 minutes of being signaled |
| Pfund et al [64];  MUAWO by Hofer et al [73] | Fixed 3 times per day (9 am, 2:30 pm, and 9pm) | Morning, afternoon, and evening | 3 | 5 | Study-provided smartphone | Not described | Not described | Not described |
| Junghaenel et al [57];  not applicable | Once at a self-determined bedtime from 6pm to 12am | Since waking up | 1 | 25 | Not described | Not described | Opt-in reminder (emails and/or text messages) | Not described |
| Bartlett and Arpin [63];  not applicable | Once at a self-determined bedtime | In the moment of assessment^d^ | 1 | 20 | Paper-based surveys (i.e., envelopes) | Not described | A reminder to complete surveys for participants who missed three consecutive daily surveys | Not described |
| Jiang et al [67];  combining Canada in study by Hoppmann et al [74] and Hong Kong data set [not presented] | Not specific | Not specific | 3-5 | 7-10 | Not described | Not described | Not described | Not described |
| Zhaoyang et al [58];  not specific name [not presented] | Mixed: (1) quasi-random basis with 2-3 hours apart for 5 times, (2) once each for morning and evening | Since the last assessment | 5 | 7 | Study-provided palmtop computer | Training on study protocol, use of the smartphone, and practice for 2 days | Not described | Responses completed within 30 minutes of being beep, and no longer than 30 min from start to finish |
| Chui et al [62];  combining ADuLTS study by Luszcz et al [75] and other source [not presented] | Quasi-random basis within each 3-hour interval for 6 times | At the moment | 6 | 7 | Not specific (electronic beeper and paper diaries) | On Day 1, researchers provided instructions on completing daily diaries and using an electronic beeper. On Day 2, researchers visited participants’ homes to check for any difficulties. | Not described | Responses completed within 2 hours |
| Heo et al [66];  not applicable | Random basis from 9am until 9pm | At the moment | 7 | 7 | Not described | Not described | Not described | Responses completed within 15 minutes of signaling |
| Rook [65];  not specific name [not presented] | Once at an end of day | During the day | 1 | 14 | Not described | In-person instruction on how to use the daily checklists at baseline | Not described | Not described |

Abbreviations: EMA, Ecological Momentary Assessment; CHART, Chicago Health and Activity Space in Real-Time; GPS, Global Positioning System; EAS, Einstein Aging Study; DEWS, Daily Experiences and Well-being Study; EAR, Electronically Activated Recorder; CCSHC, Continued Care Senior Housing Community; MUAWO, Media Use and Well-Being of Older Adults; ADuLTS, Australia Longitudinal Study of Ageing (ALSA) Daily-Life Time-Sampling.

^a^ Subsample of respondents from Wave 3 were recruited to participate in three pandemic waves spanning 4 months, each spaced approximately one month apart (hereafter referred to as “Waves 4–6”) [49].

^b^ End-of- day survey includes questions about current loneliness [43-46].

^c^ End-of- day survey includes questions about interpersonal tensions that day [35].

^d^ Loneliness questionnaire is based on the current situation.

References

15. Zhaoyang R, Scott SB, Martire LM, Sliwinski MJ. Daily social interactions related to daily performance on mobile cognitive tests among older adults. PLoS One 2021;16(8):e0256583. [doi:10.1371/journal.pone.0256583] [PMID:34437609]

26. Fingerman KL, Zhou Z, Huo M, Luong G, Birditt KS. Enduring bonds: duration and contact in close relationships in late Life. Gerontologist 2024;64(4). [doi:10.1093/geront/gnad091] [PMID:37434403]

27. Zhang S, Zhou Z, Fingerman KL, Birditt KS. Loneliness and mode of social contact in late life. J Gerontol B Psychol Sci Soc Sci 2024;79(9). [doi:10.1093/geronb/gbae115] [PMID:39192673]

28. Zhou Z, Zhang S, Kim YK, Birditt KS, Fingerman KL. Need to belong, daily social engagement, and transient loneliness in late life. Journal of Social and Personal Relationships 2023;41(1):115-136. [doi:10.1177/02654075231211617]

29. Kim YK, Fingerman KL. Daily social media use, social ties, and emotional well-being in later life. J Soc Pers Relat 2022;39(6):1794-1813. [doi:10.1177/02654075211067254] [PMID:37727534]

30. Ng YT, Huo M, Han SH, Birditt KS, Fingerman KL. Older adult's marital status, conversation frequency, and well-being in everyday life. J Gerontol B Psychol Sci Soc Sci 2022;77(3):499-512. [doi:10.1093/geronb/gbab112] [PMID:34159387]

31. Zhang S, Kim YK, Fingerman KL, Birditt KS, Charles ST. Narcissism, social experiences, and mood in late life. J Gerontol B Psychol Sci Soc Sci 2022;77(8):1442-1453. [doi:10.1093/geronb/gbac019] [PMID:35092423]

32. Fingerman KL, Ng YT, Huo M, Birditt KS, Charles ST, Zarit S. Functional limitations, social integration, and daily activities in late life. J Gerontol B Psychol Sci Soc Sci 2021;76(10):1937-1947. [doi:10.1093/geronb/gbab014] [PMID:33460446]

33. Huo M, Ng YT, Fuentecilla JL, Leger K, Charles ST. Positive encounters as a buffer: pain and sleep disturbances in older adults' everyday Lives. J Aging Health 2021;33(1-2):75-85. [doi:10.1177/0898264320958320] [PMID:32897128]

34. Ng YT, Huo M, Gleason ME, Neff LA, Charles ST, Fingerman KL. Friendships in old age: daily encounters and emotional well-being. J Gerontol B Psychol Sci Soc Sci 2021;76(3):551-562. [doi:10.1093/geronb/gbaa007] [PMID:31943103]

35. Birditt KS, Polenick CA, Luong G, Charles ST, Fingerman KL. Daily interpersonal tensions and well-being among older adults: the role of emotion regulation strategies. Psychol Aging 2020;35(4):578-590. [doi:10.1037/pag0000416] [PMID:31670541]

36. Fingerman KL, Huo M, Charles ST, Umberson DJ. Variety is the spice of late life: social integration and daily activity. J Gerontol B Psychol Sci Soc Sci 2020;75(2):377-388. [doi:10.1093/geronb/gbz007] [PMID:30783671]

37. Fuentecilla JL, Huo M, Birditt KS, Charles ST, Fingerman KL. Interpersonal tensions and pain among older adults: the mediating role of negative mood. Res Aging 2020;42(3-4):105-114. [doi:10.1177/0164027519884765] [PMID:31709931]

38. Huo M, Fuentecilla JL, Birditt KS, Fingerman KL. Does empathy have a cost? older adults and social partners experiencing problems. Gerontologist 2020;60(4):617-627. [doi:10.1093/geront/gnz065] [PMID:31152585]

39. Birditt KS, Manalel JA, Sommers H, Luong G, Fingerman KL. Better off alone: daily solitude is associated with lower negative affect in more conflictual social networks. Gerontologist 2019;59(6):1152-1161. [doi:10.1093/geront/gny060] [PMID:29924314]

40. Huo M, Fuentecilla JL, Birditt KS, Fingerman KL. Older adults' empathy and daily support exchanges. J Soc Pers Relat 2019;36(11-12):3814-3834. [doi:10.1177/0265407519837372] [PMID:31814654]

41. Ferguson G, Ali S, Pasquini G, et al. Daily and trait personality assessments’ relationship with daily experiences of stress risk and resilience factors. Journal of Research in Personality 2024;109:104452. [doi:https://doi.org/10.1016/j.jrp.2023.104452]

42. Jang H, Hill NL, Turner JR, Bratlee-Whitaker E, Jeong M, Mogle J. Poor-quality daily social encounters, daily stress, and subjective cognitive decline among older adults. Innovation in Aging 2024;8(6). [doi:10.1093/geroni/igae038]

43. Kang JE, Graham-Engeland JE, Martire LM, Almeida DM, Sliwinski MJ. Short-term coupling associations between state loneliness and cognitive performance in daily life among older adults. J Gerontol B Psychol Sci Soc Sci 2024;79(10). [doi:10.1093/geronb/gbae134] [PMID:39105303]

44. Van Bogart K, Scott SB, Harrington KD, Felt JM, Sliwinski MJ, Graham-Engeland JE. Examining the bidirectional nature of loneliness and anxiety among older adults in daily life. J Gerontol B Psychol Sci Soc Sci 2023;78(10):1676-1685. [doi:10.1093/geronb/gbad105] [PMID:37527478]

45. Van Bogart K, Engeland CG, Sliwinski MJ, et al. The association between loneliness and inflammation: findings from an older adult sample. Front Behav Neurosci 2021;15:801746. [doi:10.3389/fnbeh.2021.801746] [PMID:35087386]

46. Zhaoyang R, Harrington KD, Scott SB, Graham-Engeland JE, Sliwinski MJ. Daily social interactions and momentary loneliness: the role of trait loneliness and neuroticism. J Gerontol B Psychol Sci Soc Sci 2022;77(10):1791-1802. [doi:10.1093/geronb/gbac083] [PMID:35758315]

47. Zhaoyang R, Sliwinski MJ, Martire LM, Katz MJ, Scott SB. Features of daily social interactions that discriminate between older adults with and without mild cognitive impairment. J Gerontol B Psychol Sci Soc Sci 2021;79(4). [doi:10.1093/geronb/gbab019] [PMID:33528558]

48. Compernolle E, Finch LE, Hawkley LC, Cagney KA. Momentary loneliness among older adults: contextual differences and their moderation by gender and race/ethnicity. Soc Sci Med 2024;350:116743. [doi:10.1016/j.socscimed.2024.116743] [PMID:38522965]

49. Compernolle EL, Finch LE, Hawkley LC, Cagney KA. Home alone together: Differential links between momentary contexts and real-time loneliness among older adults from Chicago during versus before the COVID-19 pandemic. Soc Sci Med 2024;346:116744. [doi:10.1016/j.socscimed.2024.116744] [PMID:38494392]

50. Goldman AW, Compernolle EL. Personal network size and social accompaniment: protective or risk factor for momentary loneliness, and for whom? Soc Ment Health 2023;13(1):23-44. [doi:10.1177/21568693221142336] [PMID:38665906]

51. Goldman A, York Cornwell E. Stand by me: social ties and health in real-time. Socius 2023;9. [doi:10.1177/23780231231171112] [PMID:37822581]

52. Hülür G, Luo M, Macdonald B, Grünjes CE. The perceived quality of social interactions differs by modality and purpose: an event-contingent experience sampling study with older adults. Journal of Social and Personal Relationships 2024;41(4):794-821. [doi:10.1177/02654075231215269]

53. Luo M, Yordanova K, Macdonald B, Hülür G. Routineness of social interactions is associated with higher affective well-being in older adults. J Gerontol B Psychol Sci Soc Sci 2024;79(6). [doi:10.1093/geronb/gbae057] [PMID:38595036]

54. Luo M, Macdonald B, Hülür G. Not "the more the merrier": diminishing returns to daily cace-to-face social interaction frequency for well-being in older age. J Gerontol B Psychol Sci Soc Sci 2022;77(8):1431-1441. [doi:10.1093/geronb/gbac010] [PMID:35077534]

55. Luo M, Pauly T, Röcke C, Hülür G. Alternating time spent on social interactions and solitude in healthy older adults. Br J Psychol 2022;113(4):987-1008. [doi:10.1111/bjop.12586] [PMID:35957493]

56. Macdonald B, Luo M, Hülür G. Daily social interactions and well-being in older adults: The role of interaction modality. Journal of Social and Personal Relationships 2021;38(12):3566-3589. [doi:10.1177/02654075211052536]

57. Junghaenel DU, Broderick JE, Schneider S, et al. Explaining age differences in the memory-experience gap. Psychol Aging 2021;36(6):679-693. [doi:10.1037/pag0000628] [PMID:34516172]

58. Zhaoyang R, Sliwinski MJ, Martire LM, Smyth JM. Age differences in adults' daily social interactions: an ecological momentary assessment study. Psychol Aging 2018;33(4):607-618. [doi:10.1037/pag0000242] [PMID:29708385]

59. Wallimann M, Peleg S, Pauly T. Time-savoring moderates associations of solitude with depressive mood, loneliness, and somatic symptoms in older adults' daily life. Appl Psychol Health Well Being 2024;16(3):1497-1515. [doi:10.1111/aphw.12538] [PMID:38520051]

60. Badal VD, Lee EE, Daly R, et al. Dynamics of loneliness among older adults during the COVID-19 pandemic: pilot study of ecological momentary assessment with network analysis. Front Digit Health 2022;4:814179. [doi:10.3389/fdgth.2022.814179] [PMID:35199099]

61. Mann AS, Boeder J, Tse DCK, Graham L, Nakamura J. Solitary prosociality in later life: an experience sampling study. Res Aging 2022;44(9-10):724-733. [doi:10.1177/01640275211062124] [PMID:35271402]

62. Chui H, Hoppmann CA, Gerstorf D, Walker R, Luszcz MA. Social partners and momentary affect in the oldest-old: the presence of others benefits affect depending on who we are and who we are with. Dev Psychol 2014;50(3):728-740. [doi:10.1037/a0033896] [PMID:23895170]

63. Bartlett MY, Arpin SN. Gratitude and loneliness: Enhancing health and well-being in older adults. Res Aging 2019;41(8):772-793. [doi:10.1177/0164027519845354] [PMID:31043126]

64. Pfund GN, Hofer M, Allemand M, Hill PL. Being social may be purposeful in older adulthood: a measurement burst design. Am J Geriatr Psychiatry 2022;30(7):777-786. [doi:10.1016/j.jagp.2021.11.009] [PMID:34924274]

65. Rook KS. Emotional health and positive versus negative social exchanges: a daily diary analysis. Applied Developmental Science 2001;5(2):86-97. [doi:10.1207/S1532480XADS0502_4]

66. Heo J, Lee Y, Pedersen PM, McCormick BP. Flow experience in the daily lives of older adults: an analysis of the interaction between flow, individual differences, serious leisure, location, and social context. Can J Aging 2010;29(3):411-423. [doi:10.1017/s0714980810000395] [PMID:20707938]

67. Jiang D, Fung HH, Lay JC, Ashe MC, Graf P, Hoppmann CA. Everyday solitude, affective experiences, and well-being in old age: the role of culture versus immigration. Aging Ment Health 2019;23(9):1095-1104. [doi:10.1080/13607863.2018.1479836] [PMID:30621431]

68. English N, Zhao C, Brown KL, Catlett C, Cagney K. Making sense of sensor data: how local environmental conditions add value to social science research. Soc Sci Comput Rev 2022;40(1):179-194. [doi:10.1177/0894439320920601] [PMID:35400811]

69. Katz MJ, Lipton RB, Hall CB, Zimmerman ME, Sanders AE, Verghese J, Dickson DW, Derby CA. Age-specific and sex-specific prevalence and incidence of mild cognitive impairment, dementia, and alzheimer dementia in blacks and whites: a report from the einstein aging study. Alzheimer Dis Assoc Disord 2012; 26(4):335-343. [doi:10.1097/WAD.0b013e31823dbcfc] [PMID:22156756]

70. Fingerman K, Charles S, Birditt K. Inter-University Consortium for Political and Social Research distributor. Daily experiences and well-being in late life, Austin, Texas, 2016-2017. URL: https://www.icpsr.umich.edu/web/NACDA/studies/38570 [accessed 2022-11-28]

71. Jeste DV, Glorioso D, Lee EE, Daly R, Graham S, Liu J, Paredes AM, Nebeker C, Tu XM, Twamley EW, Van Patten R, Yamada Y, Depp C, Kim H. Study of independent living residents of a continuing care senior housing community: sociodemographic and clinical associations of cognitive, physical, and mental health. Am J Geriatr Psychiatry 2019; 27(9):895-907. [doi:10.1016/j.jagp.2019.04.002] [PMID:31078382]

72. Macdonald B, Hülür G. Digitalization and the social lives of older adults: protocol for a microlongitudinal study. JMIR Res Protoc 2020;9(10):e20306. [doi:10.2196/20306] [PMID:33001037]

73. Hofer M, Birrer A, Eden A, Seifert A. Daily TV use and meaning in life among older adults: the moderating role of selective and compensatory TV use. Mass Communication and Society 2022; 27(5):1006-1027. [doi:10.1080/15205436.2022.2135447]

74. Hoppmann CA, Lee JCM, Ziegelmann JP, Graf P, Khan KM, Ashe MC. Precipitation and physical activity in older adults: the moderating role of functional mobility and physical activity intentions. J Gerontol B Psychol Sci Soc Sci 2017;72(5):792-800. [doi:10.1093/geronb/gbv107] [PMID:26707498]

75. Luszcz MA, Giles LC, Anstey KJ, Browne-Yung KC, Walker RA, Windsor TD. Cohort profile: the Australian longitudinal study of ageing (ALSA). Int J Epidemiol 2016; 45(4):1054-1063. [doi:10.1093/ije/dyu196] [PMID:25468824]
