# Supplementary material for: Ecological Momentary Assessment to Measure Social Connectedness in Older Adults: Integrative Review
Source: J Med Internet Res. 2025 Jun 17;27:e66324. doi: 10.2196/66324 (PMC12214698; doi:10.2196/66324)
Supplement: Multimedia Appendix 9 [file jmir_v27i1e66324_app9.docx]

Multimedia Appendix 9. Definition and rate of compliance and adherence used in selected studies

| First author, year | Definition | Rate | Data source |
| --- | --- | --- | --- |
|  |  |  |  |
| Compernolle EL, 2024 [48] | Not described | • Of the 450 total samples, 342 were included in the analysis.  • 12,744 EMA completed in total. | CHART |
| Compernolle EL, 2024 [49] | Adherence rate: valid EMAs out of all possible EMA | • Of the 450 total samples, 110 were included in the analysis.  • 13,330 EMA completed in total; respondents completed an average of 121 EMAs across six waves (range 6-214).  • Adherence rate = 58% (valid EMAs out of all possible EMA; 13,330/23,100)  • Conditional response rate: wave 1 (82%), wave 2 (98%), wave 3 (92%), wave 4 (97%), wave 5 (95%), and wave 6 (95%). |  |
| Goldman AW, 2023 [50] | Not described | • Of the 450 total samples, 343 were included in the analysis.  • 12,359 EMA completed in total.  • 85% participate in at least one wave of EMA collection. |  |
| Goldman AW, 2023 [51] | Not described | • Of the 450 total samples, 342 were included in the analysis.  • 12,720 EMA completed in total; respondents completed an average of 37 EMAs across three waves. |  |
| Ferguson G, 2024 [41] | Not described | Not described | EAS |
| Jang H, 2024 [42] | Not described | • Analytic sample = 254  • 12,477 EMA completed in total (10,189 beep surveys and 2,288 end-of-survey)  • Compliance rate = 83.40% (beep surveys), 79.68% (end-of day surveys)  • Average 13.65 days of EMA (SD = 1.34; range=3-14 days) out of 14 days |  |
| Kang JE, 2024 [43] | Not described | • Analytic sample = 313  • Average 13.8 days of EMAs (SD = 1.18; range = 3–14 days) out of 14 days  • 17,362 EMA with valid data on state loneliness and mobile cognitive tests |  |
| Van Bogart, 2023 [44] | Not described | • Analytic sample = 317  • Drop-out = 0%  • 18,364 EMA completed in total (14,801 beeped surveys, 3,563 end-of-day surveys)  • Compliance rate = 83.38% (beeped surveys), 80.26% (end-of-day surveys) |  |
| Van Bogart K, 2022 [45] | Not described | • Of the 296 total samples, 222 were included in the analysis (total available sample of participants who provided inflammation data = 296) |  |
| Zhaoyang R, 2022 [46] | Not described | • Analytic sample = 317  • 18.363 EMA completed in total (14,801 beep surveys, 3,562 end-of-day surveys)  • Compliance rate = 83.38% (beep surveys), 80.26% (end-of-day surveys)  • Average 13.65 days of EMAs (SD = 1.32; range = 3-14 days) |  |
| Zhaoyang R, 2021 [15] | Not described | • Analytic sample = 312  • 20,224 EMA completed in total (16,156 beep surveys, 4,068 end-of-day surveys).  • Average 15.60 days of EMAs (SD = 1.49; range = 2-16 days) out of 16days |  |
| Zhaoyang R, 2021 [47] | Not described | • Analytic sample = 311  • 18,004 EMA completed in total (14,506 beep surveys, 3,498 end-of-day surveys)  • Average 13.65 days of EMAs (SD = 1.34; range = 3-14 days) out of 14days  • Compliance rate = 82.92% (beep surveys), 79.58% (end-of-day surveys)  • Compliance rates on beep surveys (85.03% vs 78.46%, t = 2.92, p = .004) and end-of-day surveys (83.89% vs 70.50%, t = 3.78, p = .000) than did participants with MCI. |  |
| Fingerman KL, 2024 [26] | Not described | • Of the 333 total samples, 313 were included in the analysis.  • 6,262 EMA completed in total. | DEWS |
| Zhang S, 2024 [27] | Not described | • Of the 333 total samples, 313 were included in the analysis.  • 6,262 EMA completed in total, respondents completed 20.01 EMA (SD = 6.03; range = 1–32), with 3.83 surveys each day (SD = 1.40; range = 1–6) out of 5-6 days  • Average 5.31 days of EMA |  |
| Zhou ZX, 2023 [28] | Not described | • Of the 333 total samples, 299 were included in the analysis.  • 6,074 EMA completed in total; respondents completed an average of 20.31 EMA surveys (SD =5.78; median = 22; range = 1-32).  • Completion rate = 71% |  |
| Kim YK, 2022 [29] | Not described | • Of the 333 total samples, 310 were included in the analysis.  • 1,617 EMA completed in total; respondents completed an average 5.2 days of EMA. |  |
| Ng YT, 2022 [30] | Not described | • Of the 333 total samples, 272 were included in the analysis.  • 15,479 EMA completed in total |  |
| Zhang S, 2022 [31] | Not described | • Of the 333 total samples, 303 were included in the analysis.  • 5,984 EMA completed in total |  |
| Fingerman KL, 2021 [32] | Not described | • Of the 333 total samples, 313 were included in the analysis.  • 6,262 EMA completed in total.  • Participants who had at least one functional limitation (n = 193) completed 3,785 EMA (average 20 EMAs) and participant who no functional limitation (n = 120) completed 2,477 EMA (average 21 EMAs) |  |
| Huo M, 2021 [33] | Not described | • Of the 333 total samples, 292 were included in the analysis.  • Completion rate = 88%  • 1,188 days of EMA reported and the average was 20.85 EMA and 4.09 days out of 4-6 days. |  |
| Ng YT, 2021 [34] | Not described | • Of the 333 total samples, 313 were included in the analysis. |  |
| Birditt KS, 2020 [35] | Not described | • Of the 333 total samples, 293 were included in the analysis.  • Average 20.85 EMAs (SD = 5.05) |  |
| Fingerman KL, 2020 [36] | Not described | • Of the 333 total samples, 313 were included in the analysis.  • 6,262 EMA completed in total; respondents completed an average 20.01 EMA. |  |
| Fuentecilla JL, 2020 [37] | Not described | • Of the 333 total samples, 313 were included in the analysis.  • 6,262 EMA completed in total.  • Average 5.29 days of EMAs (SD = 1.09) out of 5-6 days |  |
| Huo M, 2020 [38] | Not described | • Of the 333 total samples, 313 were included in the analysis.  • 6,262 EMA completed in total; respondents completed an average 20 EMA. |  |
| Birditt KS, 2019 [39] | Not described | • Of the 333 total samples, 313 were included in the analysis.  • 5,573 EMA completed in total; respondents completed and average 20 EMA (SD = 6.03). |  |
| Huo M, 2019 [40] | Not described | • Of the 333 total samples, 293 were included in the analysis.  • 1,151 days of EMA reported and an average was 4.07 out of 5-6days |  |
| Badal VD, 2022 [60] | Not described  (acceptable adherence rate > 75%) | • Analytic sample = 22  • Conditional response rate =evening (86.4%), afternoon (84.4%), morning (80.0%)  • Adherence rate = 83.9%, average 17.0 of EMAs (SD = 3.6) of 21 EMA opportunities | CCSHC study's pool |
| Hülür G, 2024 [52] | Not described | • Of the 120 total samples, 118 were included in the analysis.  • 8,902 EMA completed in total. | Specific name not mentioned |
| Luo MX, 2024 [53] | Not described | • Of the 120 total samples, 103 were included in the analysis. |  |
| Luo M, 2022 [54] | Not described | • Of the 120 total samples, 116 were included in the analysis.  • 2,198 EMA completed in total.  • Average of 18.95 days (SD = 2.79) out of 21 days.  • Compliance rate = 90% |  |
| Luo M, 2022 [55] | Not described | • Of the 120 total samples, 118 were included in the analysis.  • 11,172 valid EMA in total; respondents completed an average of 18.96 (SD = 2.77) out of 21 possible days. |  |
| Macdonald B, 2021 [56] | Not described | • Of the 120 total samples, 115 were included in the analysis.  • 2,179 EMA completed in total.  • Average 18.95 (SD = 2.81) days of EMAs out of 21 days  • Compliance rate = 90% |  |
| Wallimann M, 2024 [59] | Not described | • Of the 123 total samples, 108 were included in the analysis.  • Drop-out = 0%  • 1,428 EMA completed in total.  • Average 13.23 days (SD = 1.16; range = 9–14) out of 14 days | Not applicable |
| Mann AS, 2022 [61] | Not described | • Of the 203 total samples, 165 were included in the analysis.  • 5,501 EMA completed in total.  • Completion rate = 81.3% | Specific name not mentioned |
| Pfund GN, 2022 [64] | Not described | • Burst 1 (N = 104; 1,292 EMA observations), Burst 2 (N = 101; 1,292 EMA observations), Burst 3 (N = 93; 1,260 EMA observations) | MUAWO |
| Junghaenel DU, 2021 [57] | Not described (completion of at least 66% of the diaries for each reporting period (i.e., 3-, 7-, 14-, 21-day reporting period) | • Of the 495 total samples, 477 were included in the analysis.  • Average of 20.00 out of 21 daily diaries (SD = 1.88; range = 2-21).  • Compliance rate = 95.24% | Not applicable |
| Bartlett MY, 2019 [63] | Not described | • Of the 42 total samples, 36 were included in the analysis,  • 761 EMA completed in total (possible 840 EMA); respondents completed an average 18.63 surveys (SD = 1.84).  • Compliance rate =90% | Not applicable |
| Jiang D, 2019 [67] | Not described | • Analytic sample = 162  • In the Canadian dataset, the maximum number of EMA responses was 30. The average number of responses for each group was as follows: Caucasian Canadians 29.73 (SD = 0.71), Immigrated Caucasians 29.84 (SD = 0.71), Immigrated East Asians 29.98 (SD = 0.34).  • In the Hong Kong dataset, the maximum number of EMA responses was 35, and all participants completed the responses. | Combining two datasets from Vancouver, Canada and Hong Kong, China |
| Zhaoyang R, 2018 [58] | Not described | • Of the 214 total samples, 173 were included in the analysis.  • 5,359 EMA completed in total (possible 6,055 EMA), respondents completed an average 31 (SD = 5.89; range = 5-35).  • Completion rate = 89% | Specific name not mentioned |
| Chui H, 2014 [62] | Not described | • Of the 75 total samples, 74 were included in the analysis.  • 3,071 EMA completed in total; respondent completed an average 40.4 (SD = 4.69) out of 42. | combining ALSA and other sources |
| Heo J, 2010 [66] | Not described | • Analytic sample = 19  • 764 EMA completed in total (possible 931 EMA)  • Compliance rate = 82.1% | Not applicable |
| Rook KS, 2001 [65] | Not described | • Of the 180 total samples, 129 were included in the analysis.  • Retention rate of 71.7% at follow-up (baseline to one year later) | Specific name not mentioned |

Abbreviations: CHART, Chicago Health and Activity Space in Real-Time; EAS: Einstein Aging Study; DEWS, Daily Experiences and Well-being Study; CCSHC, Continued Care Senior Housing Community; MUAWO, Media Use and Well-Being of Older Adults; ALSA, Australia Longitudinal Study of Ageing.
